# Sociodemographic and Lifestyle Factors and Epigenetic Aging in US Young Adults: NIMHD Social Epigenomics Program

**DOI:** 10.1001/jamanetworkopen.2024.27889

**Published:** 2024-07-29

**Authors:** Kathleen Mullan Harris, Brandt Levitt, Lauren Gaydosh, Chantel Martin, Jess M. Meyer, Aura Ankita Mishra, Audrey L. Kelly, Allison E. Aiello

**Affiliations:** 1 Department of Sociology, University of North Carolina at Chapel Hill; 2 Carolina Population Center, University of North Carolina at Chapel Hill; 3 Department of Sociology, University of Texas at Austin; 4 Population Research Center, University of Texas at Austin; 5 Department of Epidemiology, Gillings School of Global Public Health, University of North Carolina at Chapel Hill; 6 Department of Population Health, University of Kansas Medical Center, Kansas City; 7 Department of Psychology, North Carolina State University, Raleigh; 8 Department of Epidemiology, Mailman School of Public Health, Columbia University, New York, New York; 9 Robert N. Butler Columbia Aging Center, Mailman School of Public Health, Columbia University, New York, New York

## Abstract

**Importance:**

Epigenetic clocks represent molecular evidence of disease risk and aging processes and have been used to identify how social and lifestyle characteristics are associated with accelerated biological aging. However, most research is based on samples of older adults who already have measurable chronic disease.

**Objective:**

To investigate whether and how sociodemographic and lifestyle characteristics are associated with biological aging in a younger adult sample across a wide array of epigenetic clock measures.

**Design, Setting, and Participants:**

This cohort study was conducted using data from the National Longitudinal Study of Adolescent to Adult Health, a US representative cohort of adolescents in grades 7 to 12 in 1994 followed up for 25 years to 2018 over 5 interview waves. Participants who provided blood samples at wave V (2016-2018) were analyzed, with samples tested for DNA methylation (DNAm) in 2021 to 2024. Data were analyzed from February 2023 to May 2024.

**Exposure:**

Sociodemographic (sex, race and ethnicity, immigrant status, socioeconomic status, and geographic location) and lifestyle (obesity status by body mass index [BMI] in categories of reference range or underweight [<25], overweight [25 to <30], obesity [30 to <40], and severe obesity [≥40]; exercise level; tobacco use; and alcohol use) characteristics were assessed.

**Main Outcome and Measure:**

Biological aging assessed from banked blood DNAm using 16 epigenetic clocks.

**Results:**

Data were analyzed from 4237 participants (mean [SD] age, 38.4 [2.0] years; percentage [SE], 51.3% [0.01] female and 48.7% [0.01] male; percentage [SE], 2.7% [<0.01] Asian or Pacific Islander, 16.7% [0.02] Black, 8.7% [0.01] Hispanic, and 71.0% [0.03] White). Sociodemographic and lifestyle factors were more often associated with biological aging in clocks trained to estimate morbidity and mortality (eg, PhenoAge, GrimAge, and DunedinPACE) than clocks trained to estimate chronological age (eg, Horvath). For example, the β for an annual income less than $25 000 vs $100 000 or more was 1.99 years (95% CI, 0.45 to 3.52 years) for PhenoAgeAA, 1.70 years (95% CI, 0.68 to 2.72 years) for GrimAgeAA, 0.33 SD (95% CI, 0.17 to 0.48 SD) for DunedinPACE, and −0.17 years (95% CI, −1.08 to 0.74 years) for Horvath1AA. Lower education, lower income, higher obesity levels, no exercise, and tobacco use were associated with faster biological aging across several clocks; associations with GrimAge were particularly robust (no college vs college or higher: β = 2.63 years; 95% CI, 1.67-3.58 years; lower vs higher annual income: <$25 000 vs ≥$100 000: β = 1.70 years; 95% CI, 0.68-2.72 years; severe obesity vs no obesity: β = 1.57 years; 95% CI, 0.51-2.63 years; no weekly exercise vs ≥5 bouts/week: β = 1.33 years; 95% CI, 0.67-1.99 years; current vs no smoking: β = 7.16 years; 95% CI, 6.25-8.07 years).

**Conclusions and Relevance:**

This study found that important social and lifestyle factors were associated with biological aging in a nationally representative cohort of younger adults. These findings suggest that molecular processes underlying disease risk may be identified in adults entering midlife before disease is manifest and inform interventions aimed at reducing social inequalities in heathy aging and longevity.

## Key Points

**Question:**

Are epigenetic clocks, measures of biological aging developed mainly on older adult samples, meaningful for younger adults, and are sociodemographic and lifestyle characteristics associated with clock measures in expected patterns found in prior aging research?

**Findings:**

This cohort study of 4237 younger adults found that sociodemographic and lifestyle factors were associated with biological aging in clocks trained to estimate morbidity and mortality, showing accelerated aging among individuals with lower levels of education and income and those with severe obesity, no weekly exercise, and tobacco use.

**Meaning:**

These findings suggest that age-related molecular processes may be identified in younger adults before disease manifests and may represent potential end points for interventions targeting social inequalities in heathy aging and longevity.

## Teaser

This cohort study investigates the association of sociodemographic and lifestyle factors with biological age as measured by epigenetic clocks among younger adults.

## Introduction

Research on aging has long documented social and demographic differentials in morbidity and mortality risk but has only recently been able to explore underlying molecular and cellular changes that accompany aging processes and shorten life. New developments in geroscience have identified biological hallmarks of aging,^1,2,3^ about which studies now collect data to examine social determinants of biological aging.^4,5,6,7,8^ In particular, epigenomic profiling has greatly increased the availability of novel indicators of biological aging in the form of epigenetic clocks,^9,10,11^ composite measures of DNA methylation (DNAm) that represent molecular evidence of disease risk and aging processes.^12,13^ Epigenetic clocks estimate epigenetic age, and the relative comparison of epigenetic age with chronological age represents epigenetic age acceleration (hereafter, *biological aging*), providing a measurement of differences in biological aging among individuals of the same calendar age.^14^ Epigenetic clock measures have been associated with many age-related diseases and mortality.^15,16,17,18,19,20,21,22,23,24,25,26^ Thus, they potentially represent useful measures for interventions intended to reduce social inequalities in healthy aging and longevity,^26^ particularly if the clocks can detect biological aging in young individuals without apparent disease.

Considerable progress has been made in measuring biological age using numerous sources of data to create epigenetic clocks.^7,27,28^ First-generation clocks were constructed as molecular estimators of chronological age.^29,30,31^ However, attention soon shifted to estimate aging outcomes beyond chronological age. Thus, second-generation epigenetic clocks were calibrated on differences in biological aging reflected by disease and mortality risks.^20,32^ Recent advances exploit longitudinal measurement of physical and cognitive function and disease risk over time to construct clocks that capture the pace of change in biological aging over time,^33,34^ representing a third generation of epigenetic clocks.

There is an increasing body of research on social and lifestyle factors associated with measured biological aging,^7,28^ providing insights for differential exposures that are associated with aging.^35,36,37,38,39^ To the extent that certain social and lifestyle factors are known to be associated with increased age-related health risks, we would expect these factors to be associated with more rapid biological aging, affirming the research value of epigenetic clocks as markers of aging, particularly in younger adults. Indeed, lower socioeconomic status in education, income, and wealth has been associated with more rapid epigenetic aging.^8,40^ Variability in biological aging by sex generally shows that males experience more rapid epigenetic aging,^28,39,41^ whereas variability by race and ethnicity is inconsistent, with positive, negative, and null differences observed in previous research comparing Black or Hispanic with White individuals.^5,9^ Among lifestyle factors, existing research shows strong and consistently positive associations of tobacco use and body mass index (BMI; calculated as weight in kilograms divided by height in meters squared) with biological aging in second- and third-generation clocks but more mixed results for alcohol use and exercise.^7,8,9,28^ Overall, results suggest that more recent, second- and third-generation clocks are more sensitive to social and environmental exposures, although more work is needed to better understand whether and how clocks capture shared or distinct aspects of aging.

Existing research examining sociodemographic factors and biological aging has notable gaps. First, many studies use a single or a few clocks, making it difficult to ascertain whether results are consistent across clocks. Second, most epigenetic research relies on small, often local, and nondiverse samples, limiting generalizability.^7,13,14,40^ Some recent studies have used large representative samples that examined a range of clocks but focused on older adults.^10,41^ In fact, most research on epigenetic aging has been based on samples of older adults^10,14,15,20,22,23,24,38,42,43,44,45^ (exceptions include studies by Aanes et al^46^ and Raffington et al^47^). Given that older adults are more likely to have chronic comorbidities, it is difficult to disentangle outcomes associated with underlying disease from those associated with sociodemographic exposures. Furthermore, as age increases, biological age may become a less reliable estimator of health outcomes due to mortality selection and increased biological heterogeneity in older age.

Our research addresses existing gaps by investigating the association of sociodemographic and lifestyle factors with biological age according to 16 DNAm measures in a diverse population of adults aged 33 to 44 years from the US representative National Longitudinal Study of Adolescent to Adult Health (Add Health). Using new methylation data with national representation of racial and ethnic, socioeconomic, and geographic groups, we contribute to the limited research on epigenetic aging in younger adults. To our knowledge, our study is one of few to examine the emergence of sociodemographic inequalities in aging before adults enter midlife across established epigenetic clock measures.

## Methods

Data in this cohort study come from Add Health, a nationally representative cohort study of US adolescents in grades 7 to 12 in 1994 who were followed up for 25 years across 5 interview waves.^44^ We use data from wave I (WI; 1994-1995) and wave V (WV; 2016-18), when the cohort was aged 33 to 44 years. During the WV survey, 5381 of 12 300 participants consented to and completed a follow-up in-person home exam, when venous blood was drawn (93.1% consent rate) for DNAm assay. After removal of samples that did not pass quality control and elimination of replicates, the DNAm sample included 4582 participants. The sample size was further reduced to 4237 participants due to missing values on sociodemographic factors. Population representation was maintained across waves and samples (eTable 1 in Supplement 1).

Methylation analysis was conducted using the Illumina Infinium chemistry.^48^ DNAm levels across approximately 850 000 CpG sites were measured using the Infinium Methylation EPIC BeadChip (Illumina, Inc). We measured β values for CpG sites across the genome according to kit protocols and filtered to remove polymorphic positions. Remaining CpG sites were restricted to a set of 30 484 CpG sites used with DNA methylation calculator^49^ and Methylcipher^50^ and Dunedin^33,34^ calculators; principal component (PC) clocks were based on 78 464 CpG sites and used code publicly available on GitHub (eAppendix 1 in Supplement 1).

Adolescent participants and their parents or caregivers provided written consent at WI; young adult participants provided consent at WV for the survey administration and epigenetic data collection. Consent obtained at WV extends to the current study. This study was approved by the institutional review board of the University of North Carolina at Chapel Hill. We followed the Strengthening the Reporting of Observational Studies in Epidemiology (STROBE) reporting guideline.

### Epigenetic Clocks

We constructed 16 epigenetic clocks when the mean (SD) cohort age was 38.4 (2.0) years at the time of venous blood draw (Table 1; eAppendix 2 in Supplement 1). Clocks are shown in order of their generation, beginning with 5 first-generation clocks: Horvath1, Horvath2, Hannum, VidalBralo, and Zhang2019. The 2 Horvath clocks were constructed across multiple tissues as a pantissue clock of chronological age and differ by the number of CpG sites on which the algorithm was based.^30,31^ The Hannum^29^ and VidalBralo^45^ clocks were trained on blood samples, and Zhang2019^51^ was trained on blood and saliva samples to estimate chronological age. We examined 3 second-generation clocks trained on disease phenotypes and mortality in estimation of epigenetic age, including Lin,^43^ PhenoAge,^20^ and GrimAge,^21^ which also incorporated smoking-associated methylation changes.

**Table 1.** Descriptive Statistics for Epigenetic Clocks and Pearson Correlation With Age (N = 4237)^a^

Type of measure	Weighted mean (SD) [range]^a^	Pearson *r*	Pearson probability
Chronological age, y	38.4 (2.0) [33.1 to 44.8]	>.99	<0.01
Clock-measured epigenetic age, y			
Horvath1	39.1 (4.3) [15.5 to 62.3]	0.42	<0.01
Horvath2	35.4 (3.5) [17.7 to 57.4]	0.58	<0.01
Hannum	31.6 (3.9) [13.2 to 47.5]	0.40	<0.01
PhenoAge	30.1 (5.7) [12.8 to 50.9]	0.32	<0.01
GrimAge	52.5 (4.6) [39.1 to 71.9]	0.39	<0.01
Lin	23.1 (5.2) [4.0 to 44.0]	0.32	<0.01
VidalBralo	54.9 (3.5) [38.2 to 77.6]	0.27	<0.01
Zhang2019	32.0 (4.1) [19.9 to 58.3]	0.62	<0.01
PC clock–measured epigenetic age, y			
PCHorvath1	45.3 (3.7) [31.6 to 66.0]	0.36	<0.01
PCHorvath2	41.2 (4.1) [26.6 to 62.5]	0.32	<0.01
PCHannum	46.1 (3.8) [31.5 to 64.8]	0.38	<0.01
PCPhenoAge	41.0 (5.3) [21.7 to 66.4]	0.34	<0.01
PCGrimAge	54.6 (3.9) [43.6 to 71.3]	0.43	<0.01
Clock-measured age acceleration, y			
Horvath1AA	0.1 (5.5) [−25.1 to 26.0]	<0.01	0.78
Horvath2AA	<0.1 (3.9) [−15.9 to 21.8]	−0.01	0.52
HannumAA	0.1 (5.0) [−25.5 to 25.0]	<0.01	0.93
PhenoAgeAA	0.1 (7.8) [−32.4 to 29.9]	0.01	0.66
GrimAgeAA	0.3 (6.2) [−15.7 to 26.6]	0.01	0.54
LinAA	0.1 (7.2) [−30.1 to 31.8]	−0.01	0.65
VidalBraloAA	−0.1 (4.8) [−23.0 to 24.0]	<0.01	0.94
Zhang2019AA	<0.1 (4.4) [−18.2 to 26.2]	−0.02	0.28
PC clock–measured age acceleration, y			
PCHorvath1AA	0.1 (5.0) [−17.9 to 31.8]	<0.01	0.97
PCHorvath2AA	<0.1 (5.5) [−20.7 to 26.9]	<0.01	0.90
PCHannumAA	0.1 (5.0) [−24.4 to 29.8]	−0.01	0.67
PCPhenoAgeAA	0.1 (7.2) [−29.1 to 32.8]	<0.01	0.89
PCGrimAgeAA	0.3 (5.2) [−14.5 to 25.4]	<0.01	0.78
Clock-measured rate of aging, SD			
Dunedin PoAm	<0.1 (1.0) [−3.9 to 4.9]	−0.01	0.52
Dunedin PACE	<0.1 (1.0) [−4.5 to 4.6]	0.08	<0.01
Zhang2017-measured risk of mortality, RR	−1.3 (0.4) [−2.6 to 0.4]	0.17	<0.01

Abbreviations: PC, principal component; RR risk ratio.

^a^ Chronological age was assessed at the wave V blood draw. Clock measures are in units of years of epigenetic (ie, biological) age. Age acceleration measures are in units of years that one’s epigenetic age was higher (positive number) or lower (negative number) compared with the epigenetic age of others with the same chronological age. Rate of aging measures are in SD units representing a faster (positive) or a slower (negative) rate of epigenetic aging (ie, biological aging) relative to the rate of chronological age over time. Risk of mortality is in units of mortality RR, with negative values indicating a lower risk and positive values indicating a higher risk.

To reduce technical variation in CpG β values on which epigenetic clocks were based, Higgins-Chen et al^52^ retrained Hannum, Horvath1, Horvath2, GrimAge, and PhenoAge clocks on PCs of CpG methylation values rather than individual CpGs, with the goal of reducing the effects of technical noise at any given individual CpG. We refer to these as PC clocks (Table 1). For each of these 13 first- and second-generation clocks, we calculated biological age by taking residuals of the clock values regressed on chronological age.

We refer to the final set of 3 clocks as third generation, which use a different unit of measurement: DunedinPoAm, Dunedin PACE, and Zhang2017. DunedinPoAm^33^ and Dunedin PACE^34^ estimate the pace of biological aging in SD units based on changes in biomarkers of organ system dysfunction, and Zhang2017 estimates a continuous risk score of all-cause mortality.^53^

### Sociodemographic and Lifestyle Characteristics

Age was a continuous measure of chronological age at WV blood draw. Participants reported sex assigned at birth at WI (female or male). We assessed differences in epigenetic aging by race and ethnicity because prior evidence reported mixed results.^5,9^ Race or ethnicity were self-identified at WV based on 1 question that asked participants, “What is your race or ethnic origin?” Response categories included American Indian or Alaska Native, Asian, Black or African American, Hispanic, Pacific Islander, White, and some other race or origin. For participants with missing data at WV, we used WI self-reports of race and ethnicity. Small sample sizes required us to combine Pacific Islander with the Asian category and American Indian or Alaska Native with the other category, although we show the full distribution in eTable 1 in Supplement 1. We measured immigrant generation at WI (first, second, or ≥third) and education (≥college, some college, or no college), annual household income (>$100 000, $50 000-$100 000, $25 000-$50 000, and <$25 000), region of residence (Northeast, West, Midwest, and South), and rural or urban residence (metropolitan, micropolitan, and small town or rural) at WV. Lifestyle measures at WV included obesity status by BMI (reference range or underweight [<25], overweight [25 to <30], obesity [30 to <40], and severe obesity [≥40]), bouts of moderate to rigorous exercise per week (≥5, 1-4, and 0), tobacco use (never, former, and current), and alcohol use (none, light [<daily and no binge drinking], and heavy [daily] or binge). See eAppendix 3 in Supplement 1 for details on variable construction.

### Statistical Analysis

We conducted descriptive statistical analysis on the 16 clocks and sample characteristics and examined correlations among epigenetic clocks, chronological age, and measures of accelerated and pace of aging. We performed weighted linear regression of accelerated biological aging on sociodemographic and lifestyle factors in bivariate and multivariable models adjusted for chronological age^54^ with and without controls for cell composition (see eMethods in Supplement 1 for model assumptions and eTable 2 in Supplement 1 for distributional attributes of clock measures). We used sampling weights in all analyses to produce national estimates^44,55,56^ and the R statistical library Survey.^57,58^ Data were analyzed using R statistical software version 4.2.1 (R Project for Statistical Computing) from February 2023 to May 2024. We conducted 2-sided tests of significance for *P* values ranging from <.001 to <.05.

## Results

We analyzed 4237 participants (mean [SD] age 38.4 [2.0] years; percentage [SE], 51.3% [0.01] female and 48.7% [0.01] male; percentage [SE], 2.7% [<0.01] Asian or Pacific Islander, 16.7% [0.02] Black, 8.7% [.01] Hispanic, and 71.0% [0.03] White). Sociodemographic and lifestyle characteristics of the sample (Table 2) reflect national representation of sex, racial and ethnic groups, and immigrant status (percentage [SE], 4.4% [0.02] first-generation and 10.0% [.01] second-generation immigrants). Most participants had at least some college education (percentage [SE], 42.7% [0.02] ≥college and 40.0% [0.01] some college), and one-third had annual household incomes greater than $100 000 (percentage [SE], 32.5% [0.02]). Reflecting current health trends, the percentage (SE) of participants with obesity (32.7% [.01]) and severe obesity (9.9% [<.01]) was high, while the percentage (SE) was 22.6% (0.01) for getting no moderate to rigorous exercise per week, 25.9% (0.01) for current smokers, and 46.8% (0.01) for heavy or binge drinking.

**Table 2.** Participant Characteristics

Characteristic	Participants, unweighted No. (weighted % [SE]) (N = 4237)
Sex	
Female	2569 (51.30 [0.01])
Male	1668 (48.70 [0.01])
Race or ethnicity	
Asian or Pacific Islander	218 (2.70 [0.006])
Black	811 (16.70 [0.023])
Hispanic	435 (8.70 [0.014])
White	2746 (71.00 [0.029])
Other^a^	27 (0.80 [0.002])
Immigrant generation	
First	200 (4.40 [0.008])
Second	551 (10.00 [0.01])
≥Third	3486 (85.70 [0.016])
Education	
≥College	2005 (42.70 [0.02])
Some college	1622 (40.00 [0.014])
No college	610 (17.30 [0.014])
Annual income, $	
>100 000	1491 (32.50 [0.015])
50 000-100 000	1403 (33.60 [0.012])
25 000-50 000	743 (18.40 [0.009])
<25 000	600 (15.50 [0.012])
Region of residence	
Northeast	438 (9.60 [0.01])
West	991 (17.70 [0.017])
Midwest	1162 (31.20 [0.027])
South	1646 (41.40 [0.021])
Rural or urban residence	
Metropolitan	3711 (85.90 [0.017])
Micropolitan	286 (7.90 [0.013])
Small town or rural	240 (6.10 [0.011])
Obesity^b^	
Reference range or underweight	1173 (26.00 [0.012])
Overweight	1304 (31.50 [0.011])
Obesity	1334 (32.70 [0.011])
Severe obesity	426 (9.90 [0.007])
Moderate to rigorous exercise, bouts/wk	
≥5	1612 (38.10 [0.012])
1-4	1656 (39.30 [0.01])
0	969 (22.60 [0.013])
Tobacco	
Never	2429 (52.30 [0.016])
Former	817 (21.90.[0.012])
Current	991 (25.90 [0.013])
Alcohol^c^	
None	231 (6.08 [0.007])
Light	2035 (47.11 [0.012])
Heavy or binge	1971 (46.81 [0.013])

^a^ The other category for race or ethnicity includes participants who identified as American Indian or Alaska Native or who checked some other race or origin.

^b^ Body mass index (BMI; calculated as weight in kilograms divided by height in meters squared) ranges for obesity categories are reference range or underweight (<25), overweight (25 to <30), obesity (30 to <40), and severe obesity (≥40).

^c^ Alcohol use is categorized as none if participants indicated they never drank alcohol, light if participants reported drinking alcohol less than daily and did not binge drink, and heavy or binge if participants reported engaging in binge drinking in the last year or drinking daily in the last month or last year.

The weighted mean and range of epigenetic ages for the various clocks (Table 1) showed considerable variation, from a mean (SD) of 23.1 (5.2) years for Lin to 54.9 (3.5) for VidalBralo for the cohort. Estimates of epigenetic age were positively correlated with chronological age. In general, first-generation clocks had higher Pearson *r* values for correlation with age except for VidalBralo (0.27), ranging from 0.40 for Hannum to 0.62 for Zhang2019. Second-generation clocks and PC clocks had moderate *r* values for correlations with chronological age, ranging from 0.32 for PhenoAge, Lin, and PCHorvath 2 to 0.43 for PCGrimAge. While DunedinPoAm was not correlated with age, DunedinPACE (Pearson *r* = 0.08) and the Zhang2017 (Pearson *r* = 0.17) risk of mortality measure were correlated with age.

**Figure 1.**
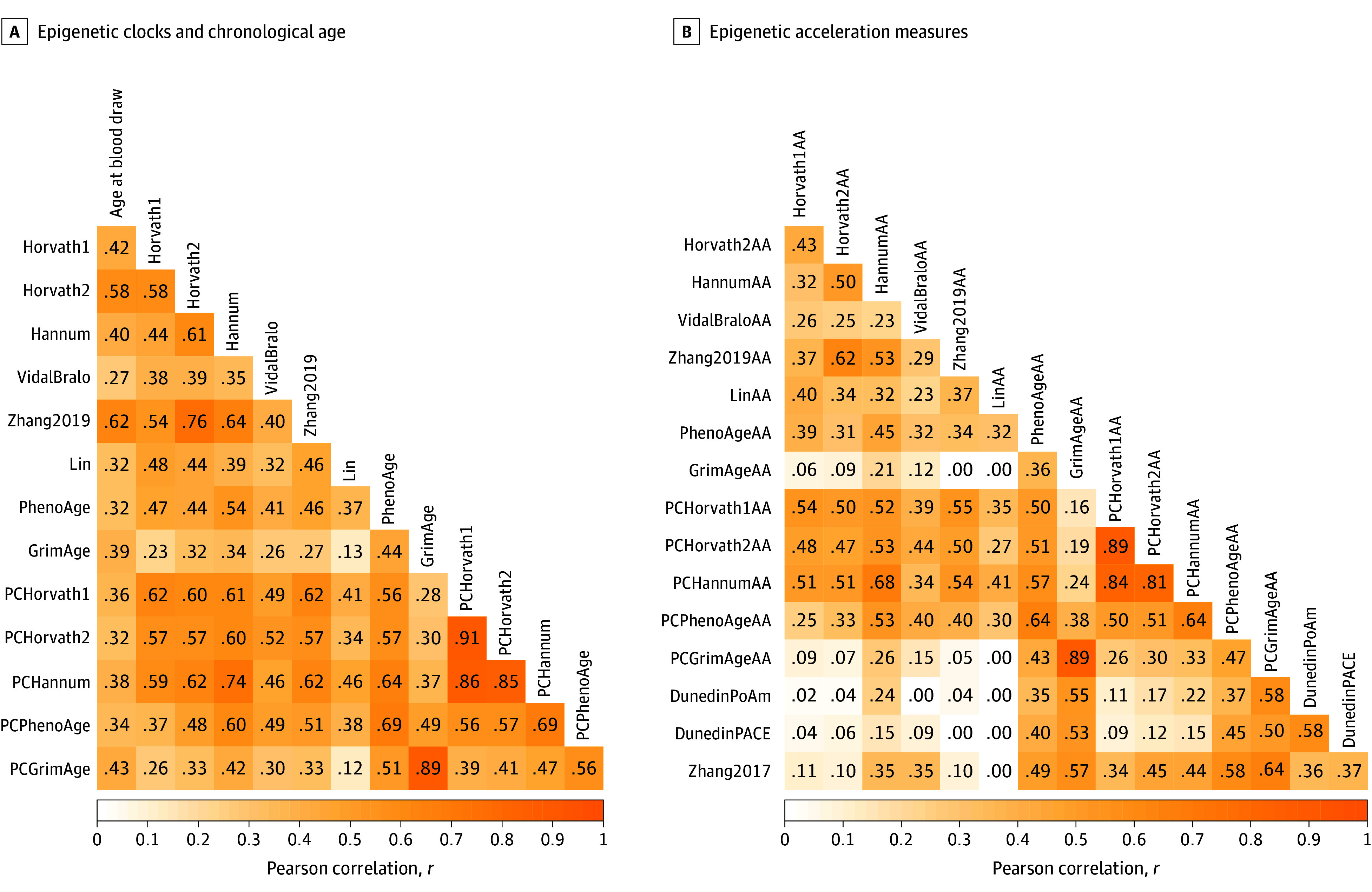
Correlations Between Epigenetic Clocks and Epigenetic Age Acceleration Correlations between epigenetic clocks and epigenetic age acceleration measures are depicted as a heat map. A, Pearson correlations among all epigenetic clocks for chronological age are presented. B, Pearson correlations among all epigenetic age acceleration measures, including third-generation rates of aging, are presented. Darker hues indicated a higher Pearson *r* correlation value.

All clocks were positively correlated with each other (Figure 1A), with the higher Pearson *r* values for correlation between first-generation clocks (except for VidalBralo) and especially among PC versions of these clocks. Among second-generation clocks, PhenoAge had a more consistent positive correlation with other clocks. Pearson *r* values for correlations among age acceleration measures were somewhat lower and less likely to reach statistical significance (Figure 1B). In general, Pearson *r* values for correlations between age acceleration measures were higher within generations of clocks compared with across generations, with the exception of PhenoAgeAA.

**Figure 2.**
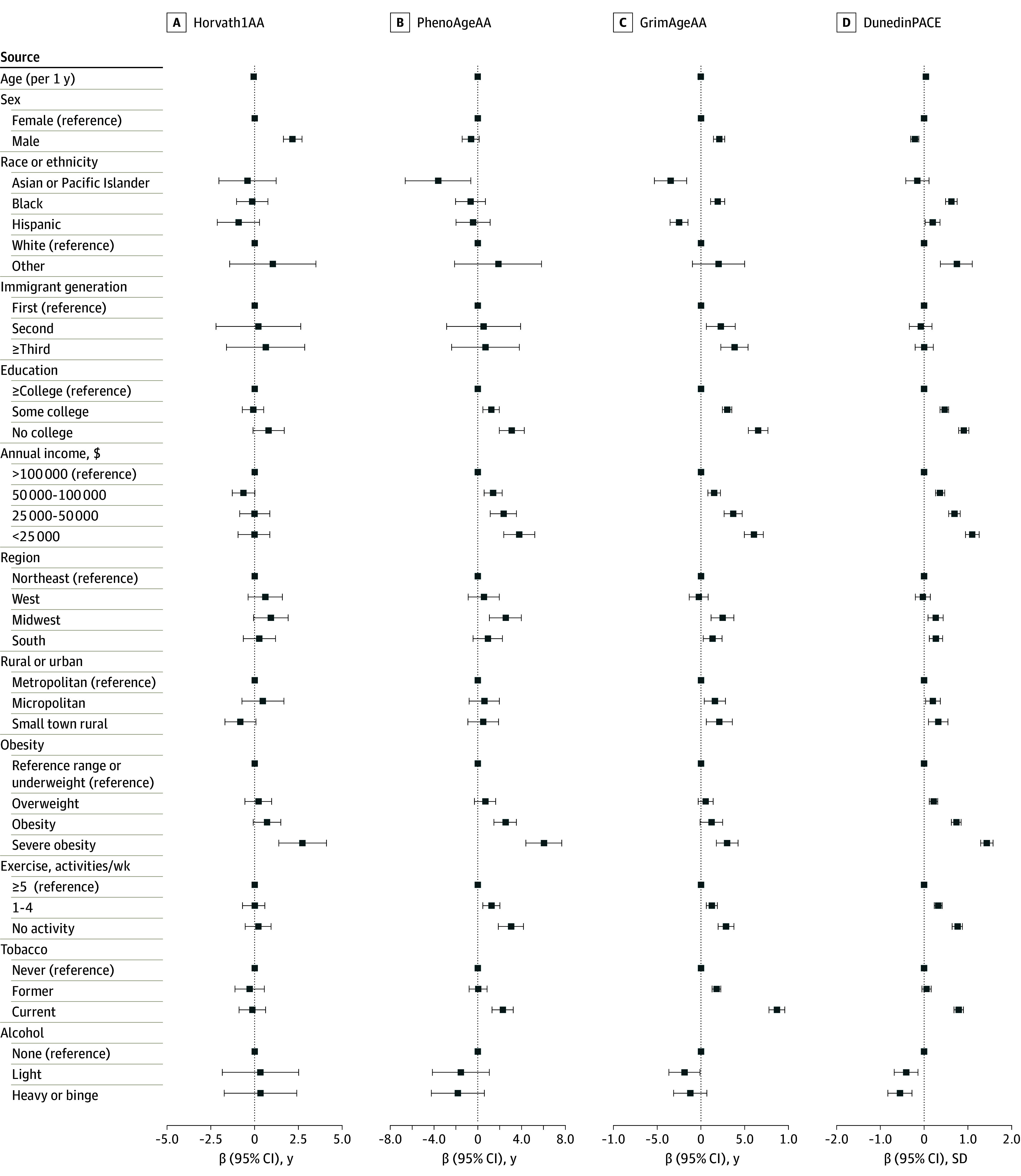
Bivariate Associations Between Sociodemographics and Epigenetic Age in Selected Clocks Weighted bivariate associations between sociodemographic characteristics and epigenetic age acceleration for selected first-, second-, and third-generation clocks are presented in a forest plot. Error bars indicate 95% CIs. Age acceleration units are expressed in years for Horvath1AA, PhenoAgeAA, and GrimAgeAA, while DunedinPACE is measured as a rate of biological aging in SD. Horvath1AA is a first-generation clock, while PhenoAgeAA and GrimAgeAA are second-generation clocks and DunedinPACE is a third-generation clock. The other category for race or ethnicity includes participants who identified as American Indian or Alaska Native or who checked some other race or origin. Alcohol use categories were none, light drinking (<daily and no binge drinking), and heavy (daily drinking) or binge drinking.

We tested bivariate associations of sociodemographic and lifestyle factors and with biological aging in which β values represent the increase (positive coefficient) or decrease (negative coefficient) in years of biological age associated with each sociodemographic category compared with the referent category. In this analysis, β values were higher among second-generation clocks PhenoAgeAA and GrimAgeAA and third-generation DunedinPACE than first-generation Horvath1AA; for example, the β for no college education vs college or more was 3.12 years (95% CI, 1.98-4.26 years) for PhenoAgeAA, 6.55 years (95% CI, 5.44-7.66 years) for GrimAgeAA, 0.90 SD (95% CI, 0.79-1.02 SD) for DunedinPACE, and 0.80 years (95% CI, −.09 to 1.69 years) for Horvath1AA (Figure 2; eTable 3 in Supplement 2). Among remaining clocks (eFigure in Supplement 1 and eTable 3 in Supplement 2), LinAA had the fewest associations among second-generation clocks, and social and lifestyle factors had high β values for associations with third-generation clocks DunedinPoAm and Zhang2017.

Overall, males had more rapid biological aging than females across most clocks. Consistent with the literature, there were mixed results for race and ethnicity; 7 first-generation clocks showed slower biological aging and 6 second- and third-generation clocks showed faster epigenetic aging among Black adults compared with White adults. More consistent results were found for Asian and Pacific Islander adults, with slower biological aging in 10 clocks, and among Hispanic adults, with slower biological aging in 5 clocks compared with White adults. Foreign-born, first-generation immigrants also had slower biological aging compared with US-born adults (eTable 3 in Supplement 2).

For the most part, second- and third-generation clocks showed expected associations, in which lower levels of education and income were associated with accelerated biological aging. A few clocks showed associations between geographic location and accelerated biological aging in the South and Midwest compared with the Northeast and in more rural locales compared with urban areas (Figure 2; eFigure 1 in Supplement 1 and eTable 3 in Supplement 2).

The lifestyle factor with the most consistent bivariate association with biological aging and highest β values was obesity status. Across 15 clocks, individuals with severe obesity (eg, BMI ≥40) experienced faster biological aging compared with those with reference range or underweight BMI status (β values ranged from 1.02 years; 95% CI, 0.22-1.83 years for Horvath2AA to 6.06 years; 95% CI, 4.42-7.69 years for PhenoAgeAA) (eTable 3 in Supplement 2); 9 clocks found faster biological aging among individuals with obesity compared with those with reference range or underweight status. Weekly exercise was also consistently and frequently associated with biological aging, showing that individuals who got less exercise had accelerated biological aging for 9 clocks. Alcohol and tobacco use results were mixed, with some clocks estimating slower aging and some faster aging with greater use. However, among second-generation clocks (except for Lin), current or former smokers had accelerated biological aging (Figure 2; eFigure 1 in Supplement 1 and eTable 3 in Supplement 2).

We tested for independent associations in a multivariable model adjusting for all sociodemographic and lifestyle characteristics (Table 3; eTable 4 in Supplement 2). Associations with the highest β values were found for second- and third-generation measures of biological aging. For example, the β for an annual income less than $25 000 vs $100 000 or more was 1.99 years (95% CI, 0.45 to 3.52 years) for PhenoAgeAA, 1.70 years (95% CI, 0.68 to 2.72 years) for GrimAgeAA, 0.33 SD (95% CI, 0.17 to 0.48 SD) for DunedinPACE, and −0.17 years (95% CI, −1.08 to 0.74 years) for Horvath1AA (Table 3). Focusing on these clocks and highlighting GrimAgeAA as an example, consistent independent associations with faster biological aging were also found for the following characteristics: lower levels of education (no college: β = 2.63 years; 95% CI, 1.67 to 3.58 years; some college: β = 0.93 years; 95% CI, 0.45 to 1.40 years) compared with college or higher, severe obesity status (β = 1.57 years; 95% CI, 0.51 to 2.63 years) compared with reference range or underweight status, lack of exercise (no bouts/wk: β = 1.33 years; 95% CI, 0.67 to 1.99 years) compared with 5 or more bouts per week, and tobacco use (current smoker: β = 7.16 years; 95% CI, 6.25 to 8.07 years) compared with never a smoker, which was a particularly high β value given that the GrimAge algorithm includes smoking pack-years. Most clocks (except PCPhenoAgeAA and DunedinPACE) showed males with faster biological aging than females (eg, GrimAgeAA: β = 1.78 years; 95% CI, 1.26 to 2.30 years). Adjusted results for race and ethnicity remained mixed as Black, Asian or Pacific Islander, and Hispanic adults had slower rates of biological aging compared with White adults across most clocks. The slower biological aging among foreign-born immigrants was independently significant only for GrimAge clocks, while HannumAA showed slower aging among US-born individuals in the third generation. Models that controlled for cell composition (eTable 5 in Supplement 2) showed similar results.

**Table 3.** Multivariate Models of Social and Demographic Characteristics and Selected Clock Estimates (N = 4237)

Variable	Horvath1AA	PhenoAgeAA	GrimAgeAA	DunedinPACE
	β (95% CI)	β (95% CI)	β (95% CI)	β (95% CI)
Age per 1 y	−0.09 (−0.25 to 0.06)	0.01 (−0.20 to 0.22)	−0.1 (−0.22 to 0.03)	0.02 (0.00 to 0.04)
Sex				
Female	[Reference]	[Reference]	[Reference]	[Reference]
Male	2.40 (1.80 to 2.99)	−0.47 (−1.27 to 0.34)	1.78 (1.26 to 2.30)	−0.23 (−0.29 to −0.16)
Race or ethnicity				
Asian or Pacific Islander	−0.18 (−2.06 to 1.71)	−3.09 (−5.90 to −0.29)	−0.98 (−2.65 to 0.68)	0.14 (-0.13 to 0.41)
Black	−0.36 (−1.17 to 0.45)	−1.93 (−3.19 to −0.68)	1.16 (0.51-1.82)	0.33 (0.22 to 0.44)
Hispanic	−0.99 (−2.51 to 0.52)	−0.84 (−2.72 to 1.04)	−1.45 (−2.43 to −0.47)	0.20 (0.04 to 0.36)
White	[Reference]	[Reference]	[Reference]	[Reference]
Other^a^	0.58 (−1.68 to 2.84)	1.31 (−2.22 to 4.83)	0.10 (−1.94 to 2.14)	0.60 (0.35 to 0.86)
Immigrant generation				
First	[Reference]	[Reference]	[Reference]	[Reference]
Second	0.35 (−1.88 to 2.57)	−0.03 (−2.73 to 2.66)	1.66 (0.36 to 2.96)	−0.07 (−0.29 to 0.15)
≥Third	0.38 (−2.01 to 2.77)	−0.84 (−3.78 to 2.11)	1.72 (0.38 to 3.07)	−0.06 (−0.28 to 0.16)
Education				
≥College	[Reference]	[Reference]	[Reference]	[Reference]
Some college	−0.12 (−0.76 to 0.51)	−0.05 (−0.77 to 0.67)	0.93 (0.45 to 1.40)	0.11 (0.02 to 0.19)
No college	0.44 (−0.50 to 1.39)	0.83 (−0.47 to 2.13)	2.63 (1.67 to 3.58)	0.30 (0.18 to 0.42)
Annual income, $				
>100 000	[Reference]	[Reference]	[Reference]	[Reference]
50 00-100 000	−0.78 (−1.40 to −0.15)	0.60 (−0.21 to 1.41)	0.28 (−0.30 to 0.86)	0.07 (−0.01 to 0.15)
25 000-50 000	−0.21 (−1.07 to 0.65)	1.13 (−0.16 to 2.41)	1.14 (0.18 to 2.10)	0.17 (0.06 to 0.28)
<25 000	−0.17 (−1.08 to 0.74)	1.99 (0.45 to 3.52)	1.70 (0.68 to 2.72)	0.33 (0.17 to 0.48)
Region				
Northeast	[Reference]	[Reference]	[Reference]	[Reference]
West	0.63 (−0.27 to 1.53)	0.90 (−0.34 to 2.14)	0.22 (−0.56 to 0.99)	0.06 (−0.04 to 0.16)
Midwest	0.80 (−0.07 to 1.68)	2.08 (0.88 to 3.29)	0.95 (0.23 to 1.67)	0.13 (0.05 to 0.22)
South	0.18 (−0.66 to 1.02)	0.66 (−0.48 to 1.79)	0 (−0.69 to 0.70)	0.10 (0.01 to 0.19)
Rural or urban				
Metropolitan	[Reference]	[Reference]	[Reference]	[Reference]
Micropolitan	0.37 (−0.67 to 1.41)	0.29 (−1.05 to 1.62)	0.46 (−0.43 to 1.36)	0.03 (−0.07 to 0.13)
Town or rural	−1.07 (−1.97 to −0.17)	−0.59 (−2.08 to 0.91)	0.26 (−0.60 to 1.12)	0.07 (−0.09 to 0.23)
Obesity^b^				
Reference range	[Reference]	[Reference]	[Reference]	[Reference]
Overweight	−0.08 (−0.85 to 0.70)	0.81 (−0.13 to 1.75)	0.11 (-0.53 to 0.75)	0.21 (0.12 to 0.30)
Obesity	0.65 (−0.11 to 1.42)	2.13 (0.98 to 3.28)	0.05 (−1.01 to 1.10)	0.58 (0.48 to 0.68)
Severe obesity	2.93 (1.69 to 4.18)	5.24 (3.55 to 6.92)	1.57 (0.51 to 2.63)	1.16 (1.04 to 1.28)
Exercise bouts/wk				
≥5	[Reference]	[Reference]	[Reference]	[Reference]
1-4	−0.05 (−0.66 to 0.56)	0.55 (-0.22 to 1.31)	0.52 (0.03 to 1.02)	0.11 (0.03 to 0.18)
0	0.29 (−0.35 to 0.92)	1.84 (0.65 to 3.04)	1.33 (0.67 to 1.99)	0.28 (0.18 to 0.38)
Tobacco				
Never	[Reference]	[Reference]	[Reference]	[Reference]
Former	−0.46 (−1.27 to 0.35)	−0.15 (−0.92 to 0.61)	1.57 (1.13 to 2.01)	0.13 (0.05 to 0.20)
Current	−0.54 (−1.33 to 0.25)	1.38 (0.32 to 2.44)	7.16 (6.25 to 8.07)	0.66 (0.55 to 0.77)
Alcohol^c^				
None	[Reference]	[Reference]	[Reference]	[Reference]
Light	0.61 (−1.46 to 2.68)	−0.81 (−3.43 to 1.82)	−0.70 (−2.01 to 0.60)	−0.12 (−0.32 to 0.09)
Heavy or binge	0.51 (−1.47 to 2.50)	−0.79 (−3.34 to 1.76)	−0.80 (−2.12 to 0.52)	−0.19 (−0.40 to 0.02)

^a^ The other category for race or ethnicity includes participants who identified as American Indian or Alaska Native or who checked some other race or origin.

^b^ Body mass index (BMI, calculated as weight in kilograms divided by height in meters squared) ranges for the obesity categories are: reference range or underweight, BMI<25; overweight, 25 ≤ BMI < 30; obesity, 30 ≤ BMI < 40; severe obesity, BMI ≥ 40.

^c^ Alcohol use is categorized as: “none” if participants indicated they never drank alcohol; “light” if participants reported drinking alcohol less than daily and did not binge drink; and “heavy or binge” if participants reported engaging in binge drinking in the last year or drinking daily in the last month or last year.

## Discussion

Based on the current literature, this cohort study estimated 16 epigenetic clocks primarily developed for older cohorts to assess their distribution, correlations with chronological age and with each other, and variability across sociodemographic and lifestyle characteristics known to estimate morbidity and mortality in prior research using the younger adult Add Health cohort. While it makes sense that most epigenetic research focuses on older adults, there is increasing recognition that molecular processes underlying disease risk begin long before overt disease is evident in chronologically older adults.^59,60^

We found that clock measures displayed a range of estimated epigenetic ages for younger adults that had moderate Pearson *r* values for correlation with chronological age and with each other. This result is consistent with prior epigenetic clock research on older populations.^9,11^ These wide-ranging results suggest that different clocks may reflect distinct aspects of aging given that they are based on the assessment of methylation at highly disparate numbers of CpG sites, trained on different populations that vary by age and race and ethnicity, and developed in different tissues.^5^

Nevertheless, many social and lifestyle factors were associated with biological aging as shown by second- and third-generation clocks in the expected direction according to prior research on inequalities in health and mortality risks^26,61,62^ even in this sample of adults about to enter midlife. In particular, GrimAge, PCGrimAge, and DunedinPACE showed accelerated aging among individuals with no or some college education compared with those with a college degree and for those at near or below poverty-level incomes compared with those with incomes greater than $100 000. Importantly and consistent with other research, severe obesity and lack of weekly exercise were also associated with faster biological aging in second- and third-generation clocks. Given that second- and third-generation clocks were trained to estimate disease and mortality risks while first-generation clocks were trained on chronological age, our findings support conclusions that the biological aging process may be underway prior to midlife and later life and that these second- and third-generation clocks are sensitive measures of this process before age-related disease comorbidities are present. Thus, our findings suggest that epigenetic clocks may represent surrogate end points in interventions designed to address social determinants of healthy aging.

We found interesting new results for immigrant status. Despite the often stressful and discriminatory contexts in which immigrants live, those with US-born status even if they had foreign-born parents in the second generation experienced faster aging, suggesting that the immigrant advantage found in much prior research remains biologically embedded.^63,64^ However, this result may be driven by the large and heterogenous Hispanic population that comprised immigrant groups given that Hispanic individuals tend to have lower biological aging for second- and third-generation clocks.

### Limitations

This study has several limitations. One potential limitation of our research is the small age range of adults in Add Health. This limitation could be related to inconsistent results we found for race and ethnicity and sex, although these findings were also consistent with prior research.^5,9,11,35,65,66^ There are differing views on whether epigenetic markers can be analyzed in pooled racial and ethnic samples, especially because many epigenetic clocks have been developed in predominantly White samples.^7,13,20,67^ It remains to be investigated whether there are varying responses to social and environmental exposures in different racial and ethnic groups.^67^ Prior research identified changes in biological aging by sex that is chronological age related (eg, females experience more rapid aging during menopause^38^), which should not affect our young adult sample. However, females also have variability in immunity over time, especially during and after pregnancy, and experience variation in autoimmunity and hormones.^68^ This variation could be related to some outcomes on which clocks are or are not trained. In addition, other social factors we did not include may explain some of the mixed sex and race and ethnicity findings.^67^

## Conclusions

There remains great promise in research using epigenetic clocks to understand underlying molecular and cellular changes that accompany aging processes and the development of chronic disease. Future epigenetic research should prioritize representative and diverse samples, such as Add Health, to evaluate whether cellular and molecular changes vary by sex and race and ethnicity so we can improve the measurement of biological aging. This cohort study further showed that cellular change in underlying disease processes was already evident in younger adults, which may inform prevention efforts. Future research should include a broader range of ages to investigate potential moderation by age or life stage to better understand when and how social inequalities in biological aging emerge across the life course.

## Notes

Supplement 1. **eAppendix 1.** Data Quality and Curation of Epigenetic Data in Add Health **eAppendix 2.** Description of Epigenetic Clock Measures **eAppendix 3.** Sociodemographic and Lifestyle Measures
eMethods.
**eFigure.** Bivariate Associations Between Sociodemographic Factors and Epigenetic Age According to Clocks **eTable 1.** Add Health Sample Distribution of Demographic Characteristics for Wave I, Wave V Full Sample, and Wave V Epigenetic Sample **eTable 2.** Univariate Statistics for DNA Methylation Epigenetic Clocks (N = 4237)

Supplement 2. **eTable 3.** Bivariate Models of Social and Demographic Characteristics and Clock Estimates (N = 4237) **eTable 4.** Multivariate Models of Social and Demographic Characteristics and Clock Estimates (N = 4237) **eTable 5.** Multivariate Models of Social and Demographic Characteristics and Clock Estimates With Cell Compositions (N = 4237)

Supplement 3.
Data Sharing Statement
