# Supplementary material for: Sociodemographic and Lifestyle Factors and Epigenetic Aging in US Young Adults: NIMHD Social Epigenomics Program
Source: JAMA Netw Open. 2024 Jul 29;7(7):e2427889. doi: 10.1001/jamanetworkopen.2024.27889 (PMC11287395; doi:10.1001/jamanetworkopen.2024.27889)
Supplement: Supplement 1. — eAppendix 1. Data Quality and Curation of Epigenetic Data in Add Health eAppendix 2. Description of Epigenetic Clock Measures eAppendix 3. Sociodemographic and Lifestyle Measures eMethods. eFigure. Bivariate Associations Between Sociodemographic Factors and Epigenetic Age According to Clocks eTable 1. Add Health Sample Distribution of Demographic Characteristics for Wave I, Wave V Full Sample, and Wave V Epigenetic Sample eTable 2. Univariate Statistics for DNA Methylation Epigenetic Clocks (N = 4237) [file jamanetwopen-e2427889-s001.pdf]

Supplemental Online Content

Harris KM, Levitt B, Gaydosh L, et al. Sociodemographic and lifestyle factors and epigenetic aging in US young adults. *JAMA Netw Open*. 2024;7(7):e2427889. doi:10.1001/jamanetworkopen.2024.27889

eAppendix 1. Data Quality and Curation of Epigenetic Data in Add Health

eAppendix 2. Description of Epigenetic Clock Measures

eAppendix 3. Sociodemographic and Lifestyle Measures

eMethods.

eFigure. Bivariate Associations Between Sociodemographic Factors and Epigenetic Age According to Clocks

eTable 1. Add Health Sample Distribution of Demographic Characteristics for Wave I, Wave V Full Sample, and Wave V Epigenetic Sample

eTable 2. Univariate Statistics for DNA Methylation Epigenetic Clocks (N = 4237)

This supplemental material has been provided by the authors to give readers additional information about their work.

© 2024 Harris KM et al. *JAMA Network Open.*

eAppendix 1: Data Quality and Curation of Epigenetic Data in Add Health

1. Purpose

Add Health (the National Longitudinal Study of Adolescent to Adult Health) is a nationally representative cohort study of U.S. adolescents in grades 7-12 in 1994 and followed for 25 years across five interview waves[1]. The Add Health epigenetic data were generated from archived blood samples collected in Wave V (2016-18) when the cohort was aged 33-44. The purpose of this appendix is to describe the provenance, quality control, and curation of the Add Health epigenetic data set.

2. Participant Sampling

Specimens were collected as part of the Wave V biomarker visit. After participation in the Wave V survey, respondents are visited by a field examiner/phlebotomist to collect physical measures, biological specimens, and a medications inventory. Venous blood (10 mL serum + 10 mL EDTA + 3 mL EDTA + 6 mL potassium oxalate / sodium fluoride + PAXgene sample), was collected via conventional phlebotomy, promptly centrifuged in the field, and securely shipped to the Laboratory for Clinical Biochemistry Research (LCBR) in Vermont for re-centrifugation, aliquoting, biomarker assay, and archival storage.[2] Archived blood samples were analyzed, cleaned and curated during 2021-2024 to generate the epigenome data as described below. The methylation subsample included 4,582 young adults with diverse social, biological, environmental, and behavioral longitudinal data from birth, GWAS, transcriptome, and biomarker data.

3. Bisulfite arrays

DNA was extracted from blood samples stored at the LCBR laboratory and quantified and quality checked using the PicoGreen dsDNA kit from Thermofisher and the Synergy4 fluorometer before being sent to the Human Genetics Center Core Laboratory at the University of Texas Health Science Center at the University of Texas, Houston (UT Houston) for methylation analysis using the Illumina Infinium chemistry. The quality of DNA was determined by gel electrophoresis and 500 nanograms of the DNA were subjected to bisulfite conversion using the EZ-96 DNAm Kit (Zymo Research Corporation; Irvine, CA, USA). DNAm levels across ~850,000 sites were measured using the Infinium MethylationEPIC BeadChip (Illumina, Inc.; San Diego, CA). Each plate included control samples, including one positive control (Universal Methylated Human DNA Standard; Zymo Research Corporation; Irvine, CA, USA), one negative control, which is human DNA that has been whole genome amplified with phi29 DNA polymerase to create an unmethylated control, and replicates that allow for evaluation of the consistency of DNAm measurements at individual CpG sites. The resulting data from the chip was read into idat files that indicate the green and red wavelength fluorescence intensity at each site on the EPIC chip and transmitted as data matrices from the UT Houston laboratory to Add Health personnel.

4. Post data collection analysis

© 2024 Harris KM et al. *JAMA Network Open.*

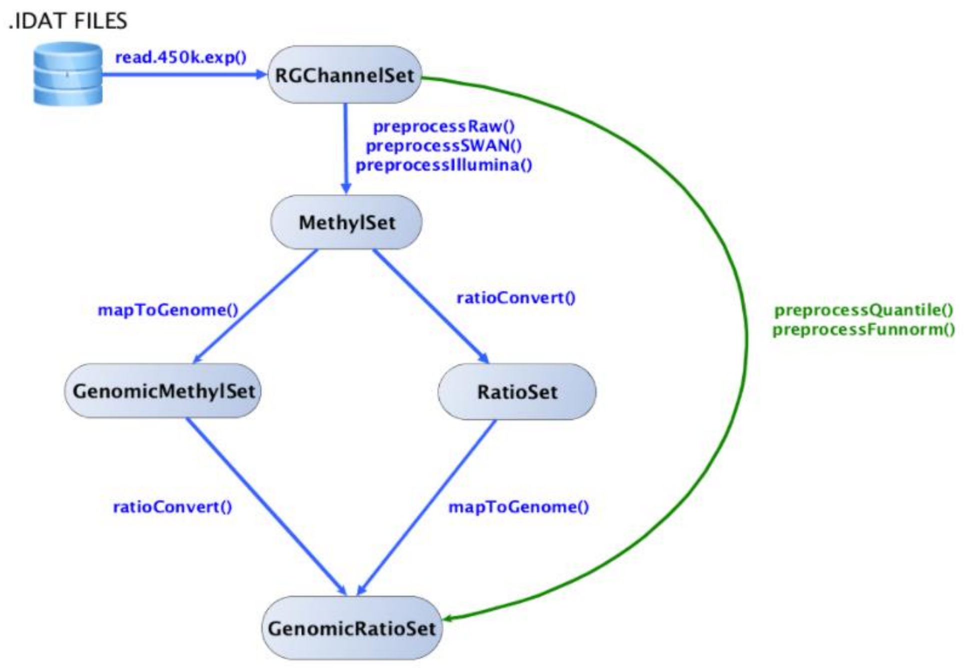

This cartoon shows the interconversions and functions that transform the various data sets that are described here.

4.1 RGChannelSet

The Minfi R bioconductor package was used to generate summarized experiment objects from the red and green fluorescence intensity matrices provided by the laboratory. The first data transformation included the creation of an RGChannelSet object using the following R code:

```
library(minfi)
library(IlluminaHumanMethylationEPICmanifest)
library(IlluminaHumanMethylationEPICanno.ilm10b4.hg19)
load('~/epigenetics1/fromUTH/EPIC.methylumi.minfi.RData')
green = rawSet@assays@data@listData$Green
red = rawSet@assays@data@listData$Red
annotation = annotation(rawSet)
rgset=RGChannelSet(Green=green, Red=red, annotation=annotation)
```

This object contains the following manifest metadata:

```
IlluminaMethylationManifest object
Annotation
  array: IlluminaHumanMethylationEPIC
Number of type I probes: 142262
Number of type II probes: 724574
Number of control probes: 635
Number of SNP type I probes: 21
Number of SNP type II probes: 38
```

© 2024 Harris KM et al. *JAMA Network Open.*

Additionally, the metadata for this summarized object is as follows:

```
class: RGChannelSet
dim: 1051539 2022
metadata(0):
assays(2): Green Red
rownames(1051539): 1600101 1600111 ... 99810990 99810992
rowData names(0):
colnames(2022): 204068280089_R03C01 204068280089_R04C01 ...
  204074220145_R02C01 204073570043_R04C01
colData names(0):
Annotation
  array: IlluminaHumanMethylationEPIC
  annotation: ilm10b4.hg19
```

4.2 Methylset

The rgset object was then converted to a methyset object that contains the unmethylated to methylated signals for each participant at each site. Further, this implements a background subtraction method that estimates background noise from the out-of-band probes and remove it for each sample separately, while the dye-bias normalization utilizes a subset of the control probes to estimate the dye bias. The mset object was generated using the following R code:

```
mset = preprocessNoob(rgset)
```

The mset object metadata is as follows:

```
class: MethylSet
dim: 865859 2022
metadata(0):
assays(2): Meth Unmeth
rownames(865859): cg18478105 cg09835024 ... cg10633746 cg12623625
rowData names(0):
colnames(2022): 204068280089_R03C01 204068280089_R04C01 ...
  204074220145_R02C01 204073570043_R04C01
colData names(0):
Annotation
  array: IlluminaHumanMethylationEPIC
  annotation: ilm10b4.hg19
Preprocessing
  Method: NA
  minfi version: NA
  Manifest version: NA
```

4.3 Genomic RatioSet

The mset object was converted to a genomic ratioset which may be used to extract betas, copy number, and m values. The function preprocessFunnorm implements a functional normalization algorithm that uses the internal control probes to infer between-array technical variation. By default, preprocessFunnorm applies the preprocessNoob function as a first step for background subtraction and uses the first two principal components of the control probes to infer the unwanted variation. This process maps the methylation signals to the genome and is implemented using the following R code:

```
grset =  preprocessFunnorm(rgset)
```

The grset object metadata is as follows:

```
class: GenomicRatioSet
dim: 865859 2022
metadata(0):
assays(2): Beta CN
rownames(865859): cg14817997 cg26928153 ... cg07587934 cg16855331
rowData names(0):
colnames(2022): 204068280089_R03C01 204068280089_R04C01 ...
  204074220145_R02C01 204073570043_R04C01
colData names(3): xMed yMed predictedSex
Annotation
  array: IlluminaHumanMethylationEPIC
  annotation: ilm10b4.hg19
Preprocessing
  Method: NA
  minfi version: NA
  Manifest version: NA
```

The betas, copy number (cn), and m values are extracted from the grset object. Some of the CpG sites overlap with short nucleotide polymorphisms and must be censored prior to betas, cn, and m value generation. This is done using the following R code:

```
grset = dropLociWithSnps(grset, snps=c("SBE","CpG"), maf=0)
beta = getBeta(grset)
m = getM(grset)
cn = getCN(grset)
```

4.4 Quality Control

4.4.1 Intensity plot

The Minfi package contains a standardized set of quality control analysis functions. The first of these functions determines if there exists an imbalance in the fluorescence intensity between methylated and unmethylated sites for any individual in the dataset. This was conducted using the following R code and resulted in the following plot:

© 2024 Harris KM et al. *JAMA Network Open.*

```
qc = getQC(mset)
plot(qc)
```

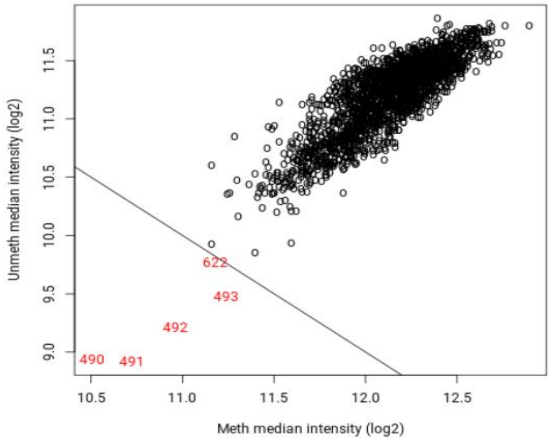

This indicates that there are 5 individuals who must be flagged because their signal intensities were below the expected rates and represent possible outliers.

4.4.2 Density plot

The second Minfi quality control function generates a density plot which analyzes if any of the samples have high levels of hemimethylation, that is, partially methylated sites. This density plot is produced using the following R code and generates the following plot:

```
densityPlot(mset)
```

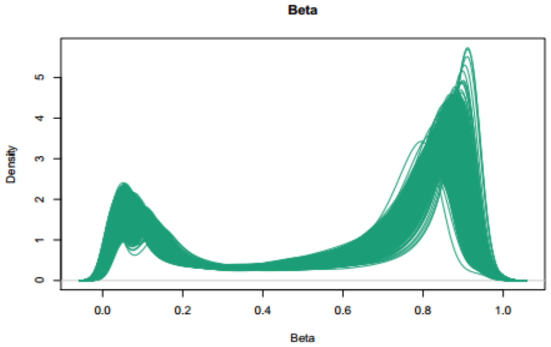

This output indicates there were no samples with an intermediate level of methylation and instead two clear peaks: one for unmethylated signal and one for methylated signal.

4.4.3 Control Strip Plot

This array contains several internal control probes that can be used to assess the quality control of different sample preparation steps (bisulfite conversion, hybridization, etc.). The values of these control probes are stored in the initial RGChannelSet and can be plotted by using the function controlStripPlot and by specifying the control probe type. This is shown for bisulfite conversion using the following R code and generates the following plot:

© 2024 Harris KM et al. *JAMA Network Open.*

controlStripPlot(rgset, controls="BISULFITE CONVERSION II")

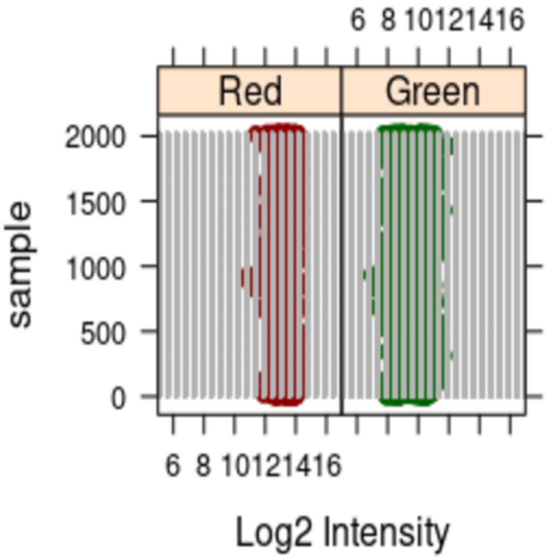

This output demonstrates that there are no outlier samples that have particularly high or low signal intensity for the green and red channels.

4.4.4 Sex check
An analysis of the signal intensity of sites on the X and Y sex chromosomes was completed and compared to recorded survey information about sex at birth. This analysis was completed using the following R code:

```
predictedSex = getSex(grset, cutoff = -2)$predictedSex
```

6 individuals were inconsistent between recorded survey responses about sex at birth and predictedSex and were censored from further analysis.

4.4.5 Multidimensional scaling
The 50000 most variable CpG sites were selected and a multidimensional analysis was conducted to identify outlier individuals. As expected, the most variable sites were primarily in the sex chromosomes and thus clustered males and females separately. The resulting plot is as follows:

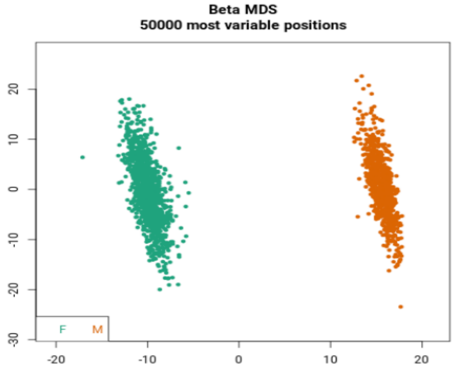

The absence of any obvious outliers and the clear clustering of males and females necessitated no more censoring of individuals.

© 2024 Harris KM et al. *JAMA Network Open.*

5 Samples and Replicates

5.1 Technical replicates

There were multiple types of embedded controls represented in this data set. The first type of embedded control was technical replicates. These were samples that were analyzed on separate chips but arose from the same sample. There were 51 such samples. The correlation between these samples was analyzed by Spearman rank correlation and presented for batch 1 in the following figure:

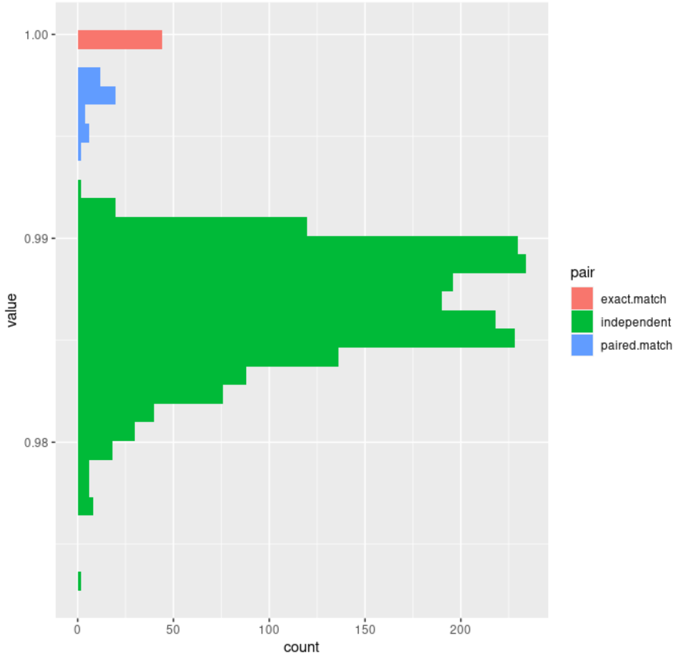

The red indicates samples compared to themselves, which, has a correlation measure of 1.0. The green indicates all comparisons between each of the 51 samples except for paired matches. Finally, blue indicates the correlation of paired samples. While the correlation rates were high for all samples, the correlation between the samples and their paired mate from a different chip were measurably higher and thus provided confidence that the inter-array variability was low.

5.2 Biological replicates

The second type of replicates present in this data set were biological replicates taken from the same participant one week apart. There may be some small changes in methylation patterns at this time scale but they should be swamped by the differences that exist between individuals. There were 200 such samples. The correlation between the first sample and the sample taken one week later as expressed in Spearman rank correlation values for batch1 are as follows:

© 2024 Harris KM et al. *JAMA Network Open.*

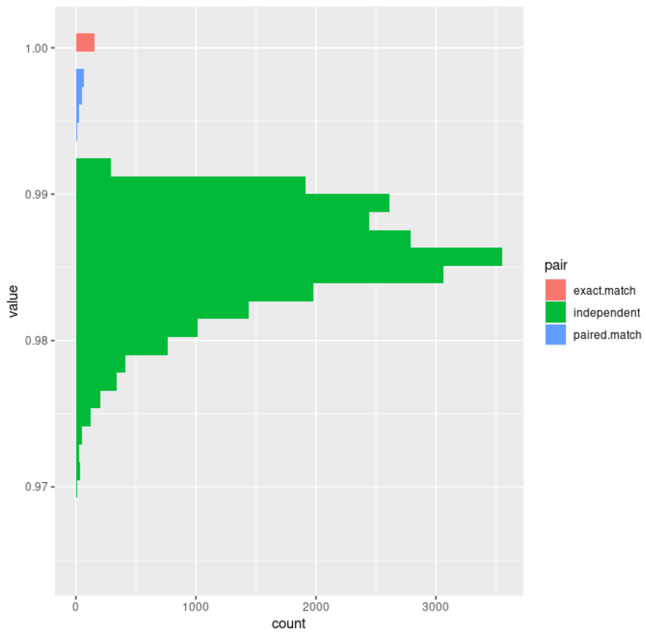

The red indicates samples compared to themselves, with a correlation measure of 1.0. The green indicates all comparisons between independent samples excluding paired matches. Finally, blue indicates the correlation of paired samples. While the correlation rates were high for all samples, the correlation between the samples and their paired mate from one week later were measurably higher and thus provided confidence that the variability in the signal was more related to inter individual differences than technical or chronological variability.

6. EPIC 850K Chip

The chip used to generate the data in this dataset is the Illumina EPIC 850K which contains 866,836 epigenetic markers including CpG sites, DNase hypersensitivity regions, SNPs, and various other probe sets. The sites covered by probes on this chip include all autosomes and both sex chromosomes. These sites include most of the sites from other common methylation arrays including the 450K chip as well as 350,000 new probes covering sites annotated by the Fantom5 and ENCODE projects as being important regulatory sites. There are two types of probe sets included in this dataset; type I probes have two separate probe sequences per CpG site (one each for methylated and unmethylated CpGs), whereas type 2 probe sets have just one probe sequence per CpG site. Type 2 probe sets are preferred because of their simplicity but type I probes are retained because they can distinguish methylation in denser regions of CpG sites. Importantly, with the addition of many more probes over previous chips, there is evidence of cross hybridization of some probe sets to related sequences elsewhere in the genome, and these require censoring as described later. Additionally, some probe sets fail to detect their targets at all and must be censored.

7. Site Filtering

The initial number of probe sets present in this data set reflect over 850,000 sites in the genome but not all are suitable for use in determining epigenetic age, allostatic load, or

© 2024 Harris KM et al. *JAMA Network Open.*

differential methylation. Some of these non-suitable sites include SNPs which may include methylation in some participants but not others as a byproduct of their nucleotide sequence. Therefore, all CpG sites overlapping with a SNP were censored from analysis using the following code in R:

```
dropLociWithSnps(grset, snps=c("SBE","CpG"), maf=0)
```

Further, some of the probes failed to detect signals for enough participants and were also censored. This was achieved using the following code in R:

```
detectionP(rgset, type = "m+u")
```

Finally, there is evidence of cross hybridization of some of the probe sets with other sites in the genome. Usage of these probes would complicate interpretation and they were also removed. The 43,000 sites identified as containing cross reactive probes[3]were censored from the final data set.

8. Sample filtering
There were 4734 samples initially analyzed for methylation patterns but several samples were removed for quality control purposes. Individual samples were removed for 3 reasons. Any individuals failing the sex check were removed from further analysis, resulting in the censoring of 1 individual. Individuals with low signal intensity from the intensity plot were flagged but not removed (n=5). Lastly, individuals with low sample volumes or concentrations such that the bisulfite conversion was impossible were not assayed. Additionally, technical and biological replicate samples were removed from the final dataset yielding 4582 samples.

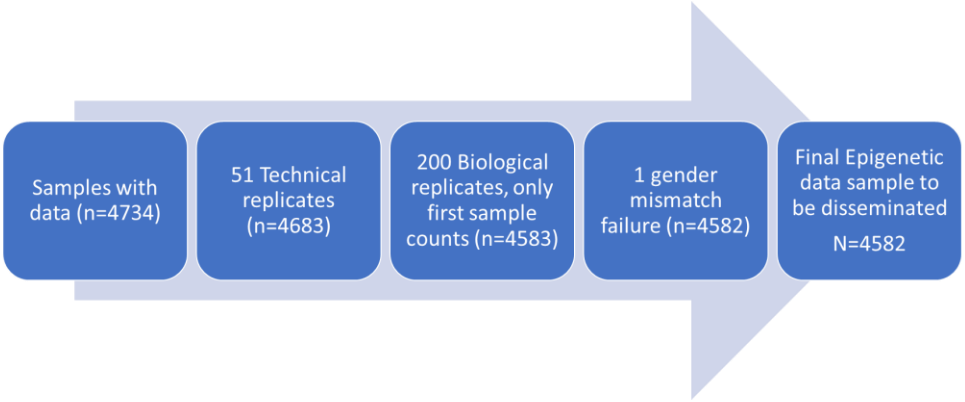

9. Batch Correction and Surrogate Variable Adjustments
The R package ComBat was used to correct for batch and the 3 batches were concatenated.

© 2024 Harris KM et al. *JAMA Network Open.*

10. Epigenetic Age Generation

Background subtracted beta values derived from the ratioset object were used with the Horvath epigenetic age clock to generate an estimated epigenetic age for each participant. The data sets were restricted to a set of 30,484 CpG sites that were relevant for the calculator and the identifier values were dummy coded to maintain data security. Finally, the data was converted to csv with Windows line endings, chunked into groups of 250 individuals, and submitted in batches to the Horvath calculator found at http://dnamage.genetics.ucla.edu/new and used with the methylCIPHER algorithm [4] https://github.com/MorganLevineLab/methylCIPHER and Dunedin calculators[5,6] to generate the epigenetic clocks.

Construction of the PC clocks required an expanded set of 78,464 CpGs, of which 1400 were missing from the processed Add Health betas. Missing CpGs were imputed in R using mean values from GSE40269 [7] according to the Levine lab code at https:hub.com/ MorganLevineLab/PC-Clocks/, and the PCs and clocks were then constructed using code from the same repository.

11. Sample Cell Counts

When a complex tissue such as whole blood is used as a sample, it is important to account for the potential differences in cell types between samples. The Minfi R package was used to calculate the relative amounts of each cell type present in the sample using the following R code:

```
cellcounts = estimateCellCounts(rgset, compositeCellType = "Blood")
```

The function analyzes CpG sites whose methylation patterns associate with one of several immune cell subsets including "Bcell", "CD4T", "CD8T", "Granulocytes", "Monocytes", and "Natural Killer Cells". The resulting values for everyone are stored as relative amounts adding up to 1 for everyone.

The resulting values are shown as follows:

© 2024 Harris KM et al. *JAMA Network Open.*

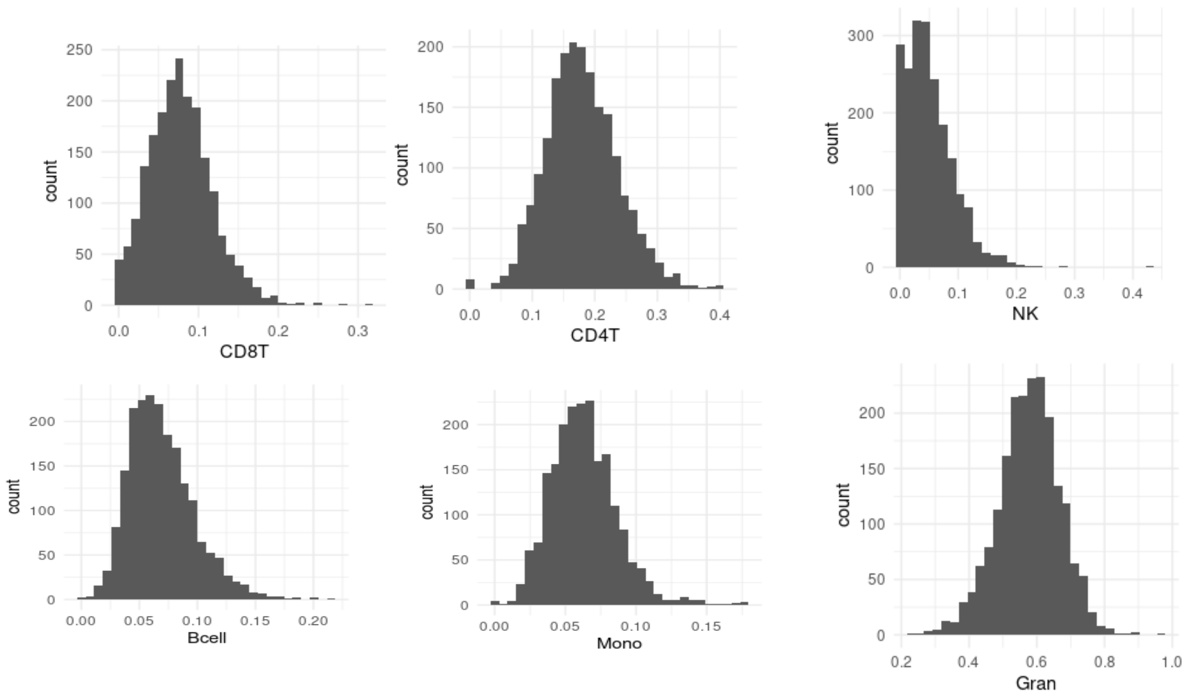

© 2024 Harris KM et al. *JAMA Network Open.*

References

1. Harris KM, Halpern CT, Whitsel EA, et al. Cohort profile: the national longitudinal study of adolescent to adult health (add health). *Int J Epidemiol*. 2019;48(5):1415-1415k. doi:10.1093/ije/dyz115

2. Measures of Glucose Homeostasis. Accessed December 21, 2023. https://cdr.lib.unc.edu/concern/scholarly_works/7w62ff69s

3. Pidsley R, Zotenko E, Peters TJ, et al. Critical evaluation of the Illumina MethylationEPIC BeadChip microarray for whole-genome DNA methylation profiling. *Genome Biol*. 2016;17(1):208. doi:10.1186/s13059-016-1066-1

4. Thrush KL, Higgins-Chen AT, Liu Z, Levine ME. R methylCIPHER: A Methylation Clock Investigational Package for Hypothesis-Driven Evaluation & Research. *BioRxiv*. July 16, 2022. doi:10.1101/2022.07.13.499978

5. Belsky DW, Caspi A, Arseneault L, et al. Quantification of the pace of biological aging in humans through a blood test, the DunedinPoAm DNA methylation algorithm. *eLife*. 2020;9. doi:10.7554/eLife.54870

6. Belsky DW, Caspi A, Corcoran DL, et al. DunedinPACE, a DNA methylation biomarker of the pace of aging. *eLife*. 2022;11. doi:10.7554/eLife.73420

7. Hannum G, Guinney J, Zhao L, et al. Genome-wide methylation profiles reveal quantitative views of human aging rates. *Mol Cell*. 2013;49(2):359-367. doi:10.1016/j.molcel.2012.10.016

© 2024 Harris KM et al. *JAMA Network Open.*

eAppendix 2: Description of Epigenetic Clock Measures

First Generation Clocks

Horvath 1

Horvath 1 is a first-generation epigenetic clock developed in 2013.[1] This clock was trained in 8,000 samples arising from 82 methylation array datasets, collectively representing 51 healthy tissues and cell types. The clock is calculated from weighted DNA methylation at 353 CpGs present in genes related to cellular survival, proliferation, and tissue development.

Horvath 1 shows strong correlation with chronological age (r=0.96-0.97). This signature is close to 0 for embryonic and pluripotent stem cells and increases with cycles of cellular replication. In 6,000 samples from cancerous tissues, Horvath 1 demonstrated substantial increases in biological age acceleration, averaging several decades older than control samples.

Horvath 2

This epigenetic clock, calculated from weighted methylation values at 391 CpGs, better measures the age of human fibroblasts and other skin cells such as keratinocytes, buccal cells, endothelial cells, lymphoblastoid cells, skin, blood, and saliva samples compared to its predecessor.[2] The improved clock correlates with chronological age in neurons, glia, brain, liver and bone samples. In contrast to the Horvath 1 signature, Horvath 2 predicts biological age acceleration in pathologies related to progeria such as Werner Syndrome. This signature was trained on epigenetic datasets from 10 studies in which the participants had median ages of 0-69 years old.

This clock shares 45 CpGs with the blood-based clock from Hannum[3] and 60 CpGs with Horvath 1; however, the age acceleration only shows moderate correlations with these two other clocks. Horvath 2 correlates well with cellular passage number but does not show any relationship with telomere length in blood samples.

Hannum

Hannum's epigenetic clock is a blood-based age estimator, calculated from weighted DNA methylation at 71 CpGs.[3] Hannum et al. developed this clock based on the whole blood of 656 humans at ages 19 to 101 at UCSF, USC, and West China Hospital. Hannum shows a strong correlation with chronological age (r=0.96) and the rate of DNAm aging is influenced by gender and genetic variants including meQTLs. While this signature was trained on blood samples, it also performs well in breast, kidney, lung and skin samples. Hannum also correlates with gene expression of age-related genes including those involved in developmental biology and DNA repair pathways.

VidalBralo

The Vidal-Bralo et al. clock[4] is calculated from weighted methylation values of 8 CpGs, that were selected as the most informative CpGs in a training set of whole blood

© 2024 Harris KM et al. *JAMA Network Open.*

samples from 390 healthy individuals from the United Kingdom Ovarian Cancer Study, Human Aging-associated DNA Hypermethylation at Bivalent Chromatin Domains dataset, and Genome-wide Analysis of Autosomal Sex Differences in Human DNA Methylome dataset. The training population for this signature included individuals aged 20-78 with a mean age of 61.2. Importantly, 96.7% of the training set participants were female. This clock targeted older adults to calibrate DNAm age more accurately among adults compared to pre-adolescents. Results were not significantly influenced by sex, smoking, or variation in blood cell subpopulations. The Vidal-Bralo signature correlates with Horvath 1, Hannum, and Weidner signatures.

Zhang2019
The Zhang epigenetic clock was constructed from 12,661 blood and saliva samples from the Lothian Birth Cohort of 1921 and 1936 Study.[5] The training set population for this signature had a mean age of 86 (Wave 3) when blood was collected. The signature is comprised of 514 CpG probes identified by an elastic net regression on chronological age. While this clock predicts a biological age that correlates with chronological age, it does not correlate with all-cause mortality in validations using subsets of the training population.

Second Generation Clocks

Lin
The Lin epigenetic clock measures a relative risk of all-cause mortality.[6] This 99 CpG model was originally trained on DNAm profiles of normal blood samples (n=446) with mortality data from the Lothian Birth Cohort 1921 Study. The mean age for participants in the training dataset was 79 years old and the authors note that it systematically overinflates the ages of younger samples. This signature correlates with chronological age and is associated with malignancy of tumor cells. This signature also correlates with telomere length.

PhenoAge
The PhenoAge clock predicts a phenotypic age trained on 9 clinical biomarkers (albumin, creatinine, serum glucose, CRP, lymphocyte percent, mean red cell volume, red cell distribution width, alkaline phosphatase, white blood cell count) which estimates an individual's mortality risk.[7] The PhenoAge clock is calculated from weighted methylation values at 513 CpGs in whole blood and was trained in the NHANES III study of individuals >=20 years old (n=9926). Levine et al. found that this clock correlated with age and predicted mortality better than first generation clocks. PhenoAge predicts risk of multiple aging outcomes such as mortality, cancer, healthspan, physical function and Alzheimer's disease and shows high correlation with biomarkers such as high CRP, glucose, triglycerides waist-to-hip ratio and low HDL cholesterol. PhenoAge was validated on data from several studies including NHANES IV, InCHIANTI, Jackson Heart Study, Women's Health Initiative, Framingham Heart Study, and the Normative Aging Study.

© 2024 Harris KM et al. *JAMA Network Open.*

GrimAge

GrimAge predicts a phenotypic age trained on 7 surrogates of plasma proteins and smoking pack years.[8] First, authors defined surrogate biomarkers of physiological risk and stress factors with plasma proteins (including adrenomedullin, CRP, plasminogen activation inhibitor 1 (PAI-1) and growth differentiation factor 15 (GDF15)) and DNAm-based estimator of smoking pack-years. Then, time-to-death was regressed on these biomarkers and an estimator of smoking years to estimate a composite biomarker of lifespan. Lu et al. report that the rate of GrimAge-based aging has predictive ability for time to death, coronary heart disease, cancer and age-related conditions. The training data for this signature comes from the Framingham Heart Study including n=2356 individuals with mean ages of 66 years. 1030 CpGs associate with the 7 composite scores. This signature was validated on n=7375 participants from Inchianti, JHS, WHI and FHS. The signature is robust to adjustment for bmi, education, alcohol, smoking, diabetes, cancer, and hypertension. However, it fails to correlate with telomere length.

PC Clocks

There are many potential sources of technical variation in CpG beta values on which epigenetic clocks are based, leading to low reliability of CpG beta values (or M values), which can result in low intraclass correlation coefficients (ICCs) for epigenetic clock values in replicate samples. In an effort to increase the epigenetic aging signal to technical noise ratio of frequently used clocks, Higgins-Chen and colleagues retrained the Hannum, Horvath1, Horvath2, GrimAge, and PhenoAge clocks on PCs of CpG methylation values rather than on individual CpGs.[9] This hypothetically increases the number of CpGs contributing to an epigenetic age calculation, thus reducing the effects of technical noise at any given individual CpG.

A unique set of methylation PCs was calculated for each clock, starting with a standard set of 78,464 CpGs that overlap across all original training datasets for the clocks and that are present on both the 450K and EPIC arrays (see supplementary table 6 of Higgins-Chen et al for details). PCA on the separate training datasets resulted in 655 PCs for Hannum, 4,280 PCs for Horvath1, 894 PCs for Horvath2, 3,934 PCs for GrimAge, and 4,504 PCs for PhenoAge. Elastic net regression was then used to train these PCs to predict either the original training target (for PCPhenoAge, which was trained on the same phenotypic age biomarker score used by Levine et al) or the original epigenetic clock age (for PC Hannum, PC Horvath 1, PC Horvath 2, and PC GrimAge) if complete training data from the original clock was not available. Thus, the PC versions of these clocks predict the same outcomes (chronological age, biological age, mortality, etc.) as their non-PC clocks. Elastic net regression retained 390 PCs for PCHannum, 121 PCs for PCHorvath1, 140 PCs for PCHorvath2, 1,936 PCs for PCGrimAge, and 652 PCs for PCPhenoAge. All PC clocks were subsequently validated in independent testing datasets and showed high correlation with the original clocks in training and testing datasets. The PC clocks were also tested on technical replicates

© 2024 Harris KM et al. *JAMA Network Open.*

and show improved ICC values compared to their original (non-PC) clocks for both clock ages and age acceleration (clock age regressed on chronological age).

PC Hannum, PCHorvath1, PCHorvath2, PCGrimAge, and PCPhenoAge were calculated in R using code available on GitHub at https://github.com/MorganLevineLab/PC-Clocks/.

Third Generation Clocks

DunedinPoAm
The DunedinPoAm epigenetic signature measures the pace of aging.[10] This signature modeled the change over time of 18 biomarkers of organ system dysfunction in n=954 participants of the Dunedin Study. The age of the mostly White participants in this training set was 26 years old at the time of the first blood collection and 38 years at the time of the second blood collection. An elastic net regression was used to compute weights for 46 CpGs. The signature correlates modestly with Horvath, PhenoAge, and Hannum signatures but outperforms all of them as a proxy of self-rated health. The signature is Z-scaled such that the mean value of the analytical sample set is 0 and negative values indicate a reduced rate of aging compared to positive values.

DunedinPACE
The DunedinPACE epigenetic signature updates the DunedinPoAm epigenetic signature using the same approach, but this iteration includes 19 indicators of organ system integrity at 4 time points including a timepoint in which the oldest participants are 45 years old.[11] DunedinPACE correlates with the DunedinPoAm (r = 0.57). This signature shows robust ability for replication. Like the DunedinPoAm signature, this signature is Z scaled such that negative values indicate slowed aging.

Zhang2017
Zhang et al. epigenetic signature is based on 10 CpGs that showed a strong association with all-cause mortality,[12] which was selected from replicated results (58 out of 11,063 CpGs with FDR<0.05) from an epigenome-wide association study (EWAS) for all-cause mortality. This epigenetic signature specifically identifies those with increased risk of death by cancer and cardiovascular disease. The training set for this signature comes from the ESTHER study and includes 406 deceased participants with blood sampled at ages 50-75 years old. Although Zhang2017 may not strictly constitute a third generation of epigenetic clock, we group presentation of results for this clock with DunedinPoAm and DunedinPACE because, similar to those clocks, its unit of measurement is not epigenetic age in years, but rather, in this case, the risk of mortality.

© 2024 Harris KM et al. *JAMA Network Open.*

References

1. Horvath S. DNA methylation age of human tissues and cell types. *Genome Biol*. 2013;14(10):R115. doi:10.1186/gb-2013-14-10-r115

2. Horvath S, Raj K. DNA methylation-based biomarkers and the epigenetic clock theory of ageing. *Nat Rev Genet*. 2018;19(6):371-384. doi:10.1038/s41576-018-0004-3

3. Hannum G, Guinney J, Zhao L, et al. Genome-wide methylation profiles reveal quantitative views of human aging rates. *Mol Cell*. 2013;49(2):359-367. doi:10.1016/j.molcel.2012.10.016

4. Vidal-Bralo L, Lopez-Golan Y, Gonzalez A. Simplified assay for epigenetic age estimation in whole blood of adults. *Front Genet*. 2016;7:126. doi:10.3389/fgene.2016.00126

5. Zhang S, Wu Z, Xie J, Yang Y, Wang L, Qiu H. DNA methylation exploration for ARDS: a multi-omics and multi-microarray interrelated analysis. *J Transl Med*. 2019;17(1):345. doi:10.1186/s12967-019-2090-1

6. Lin Q, Weidner CI, Costa IG, et al. DNA methylation levels at individual age-associated CpG sites can be indicative for life expectancy. *Aging (Albany NY)*. 2016;8(2):394-401. doi:10.18632/aging.100908

7. Levine ME, Lu AT, Quach A, et al. An epigenetic biomarker of aging for lifespan and healthspan. *Aging (Albany NY)*. 2018;10(4):573-591. doi:10.18632/aging.101414

8. Lu AT, Seeboth A, Tsai P-C, et al. DNA methylation-based estimator of telomere length. *Aging (Albany NY)*. 2019;11(16):5895-5923. doi:10.18632/aging.102173

9. Higgins-Chen AT, Thrush KL, Wang Y, et al. A computational solution for bolstering reliability of epigenetic clocks: Implications for clinical trials and longitudinal tracking. *Nat Aging*. 2022;2(7):644-661. doi:10.1038/s43587-022-00248-2

10. Belsky DW, Caspi A, Arseneault L, et al. Quantification of the pace of biological aging in humans through a blood test, the DunedinPoAm DNA methylation algorithm. *eLife*. 2020;9. doi:10.7554/eLife.54870

11. Belsky DW, Caspi A, Corcoran DL, et al. DunedinPACE, a DNA methylation biomarker of the pace of aging. *eLife*. 2022;11. doi:10.7554/eLife.73420

© 2024 Harris KM et al. *JAMA Network Open.*

12. Zhang Y, Hapala J, Brenner H, Wagner W. Individual CpG sites that are associated with age and life expectancy become hypomethylated upon aging. *Clin Epigenetics*. 2017;9:9. doi:10.1186/s13148-017-0315-9

13. Anthropometrics. Accessed December 22, 2023. https://cdr.lib.unc.edu/concern/scholarly_works/9880vw44f

14. Clinical guidelines on the identification, evaluation, and treatment of overweight and obesity in adults. *WMJ*. 1998;97(9):20-21, 24.

eAppendix 3: Sociodemographic and Lifestyle measures

Fixed demographic characteristics come from Wave I (race or ethnicity, sex, and immigrant status) whereas other sociodemographic and lifestyle covariates measured at the time blood was collected for DNAm analysis come from Wave V (e.g. education, income, region of residence, rural/urban residence, obesity, tobacco use, alcohol use).

Age at blood draw was calculated in years based on birth year (collected and validated across all waves of data) and calendar month and year of the in-home exam in Wave V when venous blood was drawn for methylation data. Sex assigned at birth was reported at Wave I but also cross-checked and validated across all Add Health waves of data.

Race or ethnicity was self-identified by participants at Waves I and V (to fill in for missing values). At Wave V race or ethnicity was asked in one question (e.g., What is your race or ethnic origin?). At Wave I race or ethnic origin is derived from two questions (Are you of Hispanic or Latino origin?; all those who indicate they are Hispanic are categorized as Hispanic and then race is assigned based on second question, What is your race?). We use Wave V race or ethnicity but fill in the few missing with Wave I reports. Participants may check multiple identities and those that do are then asked to report the race or ethnicity with which they most strongly identify, including Asian, Black, Hispanic, American Indian or Alaska Native, Other, Pacific Islander, or White. Participants who responded as “Other” did not identify with any race or ethnic categories or identified as multi-racial only. Although we display the full distribution on race or ethnicity in eTable 1, small sample sizes and the risk of deductive disclosure (per contractual agreement with Add Health) required us to combine Pacific Islander with Asian and American Indian or Alaska Native with the “other” category” in the manuscript tables.

Immigrant generation was determined at Wave I based on the participant and their parent surveys. Generation 1 are those who were foreign-born with foreign-born parents; generation 2 are those who were U.S.-born with one or two foreign-born parents; and generation 3+ are those who were U.S.-born with both U.S.-born parents.

We used categorical responses to the Wave V survey question on the highest level of education and collapsed further into three categories of college or more; some college; and no college. Participants were asked what their total household income was before taxes and deductions in the last calendar year for all household members who contribute to household expenses; responses categories were provided in 13 brackets to reduce non-response. We further collapsed categories into the following four income levels: over $100,000; $49,999-$100,000; $24,999-$50,000; and $25,000 or less. The census region in which the participant lived at Wave V was coded from their address (Northeast, West, Midwest, South). Rural/urban residence patterns were derived from Wave V Contextual data based on the “Rural Urban Commuting Area” (RUCA) codes from 2010. Three mutually exclusive categories were constructed from participants’ description of the area in which their residence was located: metropolitan; micropolitan, small town or rural.

© 2024 Harris KM et al. *JAMA Network Open.*

Bouts of exercise per week were determined from five items in the Wave V survey that inquired about the number of times in the past week the participant performed the following forms of exercise respectively, aerobic activities, bicycling, gym activities, individual sports, or golf. The number of each of these types of activity was summed and categorized as: 0, 1-4 times per week, or 5+ times per week. At Wave V participants were asked whether they had ever smoked and whether they were current smokers. From these questions we categorized tobacco use as never; former and current. To categorize participants according to their usage of alcohol at Wave V, we first used the question whether they had ever drank alcohol, and if they answered that they had not, they were categorized as “None”. If participants said that they had ever drank, we then used questions on the number of days drank last month, days drank last year, and frequency of binge-drinking. If the participant engaged in binge drinking in the last year or had reported drinking daily in the last month or last year, they were categorized as “Heavy/Binge” and all other participants that drank less than daily and did not binge drink were categorized as “Light”.

We constructed our measure of obesity status based on body mass index (BMI) constructed from measure height and weight at Wave V in the in-home exam. At Wave V, field staff measured height in cm from shoeless participants standing on uncarpeted floors and recorded weight to the nearest 0.1 kg. BMI was computed as kg/m^2 and categorized obesity status as normal or underweight (BMI<25); overweight (25≤BMI<30), obese (30≤BMI<40), and severely obese (BMI≥40).[13,14]

Overall, there were few missing values on the sociodemographic and lifestyle characteristics. Those covariates with missing values included Wave V income (N=54), Wave I immigrant generation (N=60), Wave V obesity status (N=34), Wave V smoker status (N=27), Wave V alcohol use (N=12), and Wave V education (N=1) (there is some overlap such that some participants have multiple covariates with missing values). In an analysis (not shown) we compared the sample statistics for the epigenetic clock measures in the analytic sample (which drops those missing on covariates, N=4237) with the sample statistics for the complete epigenetic sample (N=4564.The results indicated that the mean, SD, and Pearson correlation estimates were nearly identical and where there are any differences, they were within .1-.2 decimal points.

© 2024 Harris KM et al. *JAMA Network Open.*

4. eMethods

We use "sampling weights" in all analyses as recommended by the Add Health study to adjust for 1) unequal probabilities (oversampling) of selection into the sample according to specific individual characteristics as defined by the Add Health design (e.g., race and ethnicity, disability status, genetic relationship to siblings, etc.) and 2) differential attrition over time.[1-3] Add Health recommends using sampling weights in all analyses to produce representative estimates of the U.S. population of adolescents in grades 7-12 in 1994 who are followed through young adulthood to ages 33-43 in 2016-18 (i.e., Wave V)—the national population that Add Health represents.

Like all prospective longitudinal studies, Add Health has experienced attrition from the original Wave I sample over the past 25 years.[4] In addition, the epigenetic sample is a subsample of the Wave V sample in which participants consented to an in-home physical exam and blood draw. To address whether the Wave V and epigenetic samples represent the same national population in Add Health that was originally sampled at Wave I, we compared the weighted distribution of respondents at Wave I, Wave V full sample, and Wave V Epigenetic sample according to their demographic characteristics at Wave I (sex, race/ethnicity, and immigrant status) (**eTable 1**). This analysis shows the N of participants and the weighted distribution on Wave I characteristics in each wave and subsample. The sampling weights adjust for the complex sampling design and differential attrition across waves/samples and ensure that bias is not introduced into parameter estimates in all analyses.

Results show that while there is some attrition according to factors for which attrition is commonly found in most studies (e.g., minoritized racial or ethnic identities, males, and native-born)[4], the weighted percentage distribution by race/ethnicity, sex, and immigrant status are relatively similar across these samples. The Wave V sample and Wave V epigenetic sample have slightly more White participants (1.5% and 2.7%, respectively) than the Wave I sample. Common to most surveys, males have higher attrition rates, though the differences are also quite minor (at most, a 2% point difference). Attrition is also higher among the foreign-born population who often return to their country of origin, but even there, the distribution is only different in the epigenetic sample by 1% point. These results match Add Health's relatively high response rates, low attrition, and findings of minimal bias associated with attrition.[1] For more information regarding the Wave V sample design and sampling weights see Harris et al. (2019).[2]

We use regression models to estimate the weighted associations of sociodemographic and lifestyle characteristics with the various epigenetic clocks. To confirm the appropriateness of regression parameterization, we examined the univariate percentile distribution of the various clock outcomes (**eTable 2**). These distributions suggest a normal distribution assumption of the clock outcomes is relatively valid and justifies our use of regression models, especially for our main outcomes, the accelerated and rates of biological aging measures, as well as the risk of mortality. As described above in eAppendix 2 (Description of Epigenetic Clock Measures) and shown in Table 1 and

© 2024 Harris KM et al. *JAMA Network Open.*

eTable 2, different algorithms produce quite different predicted ages, resulting in different "accelerations."

References

1. Harris KM, Halpern CT, Whitsel EA, et al. Cohort profile: the national longitudinal study of adolescent to adult health (Add Health). Int J Epidemiol. 2019;48(5):1415-1415k. doi:10.1093/ije/dyz115

2. Harris KM, Halpern CT, Biemer P, Liao D, Dean SC. Add Health Wave V Documentation: Sampling and Mixed-Mode Survey Design, 2019; Available from: http://www.cpc.unc.edu/projects/addhealth/documentation/guides/https://doi.org/10.17615/v1vy-a536

3. Biemer, P.P., K.M. Harris, D. Liao, B.J. Burke, and C.T. Halpern. 2021. Transitioning a panel survey from in-person to predominantly web data collection: Results and lessons learned. Journal of the Royal Statistical Society. https://doi.org/10.1111/rssa.12750

4. Groves, R.M., Couper, M.P. 2012. *Nonresponse in Household Interview Surveys. Wiley Series in Survey Methodology*, John Wiley & Sons.

© 2024 Harris KM et al. *JAMA Network Open.*

eFigure. Bivariate associations between epigenetic age associations and sociodemographic covariates.

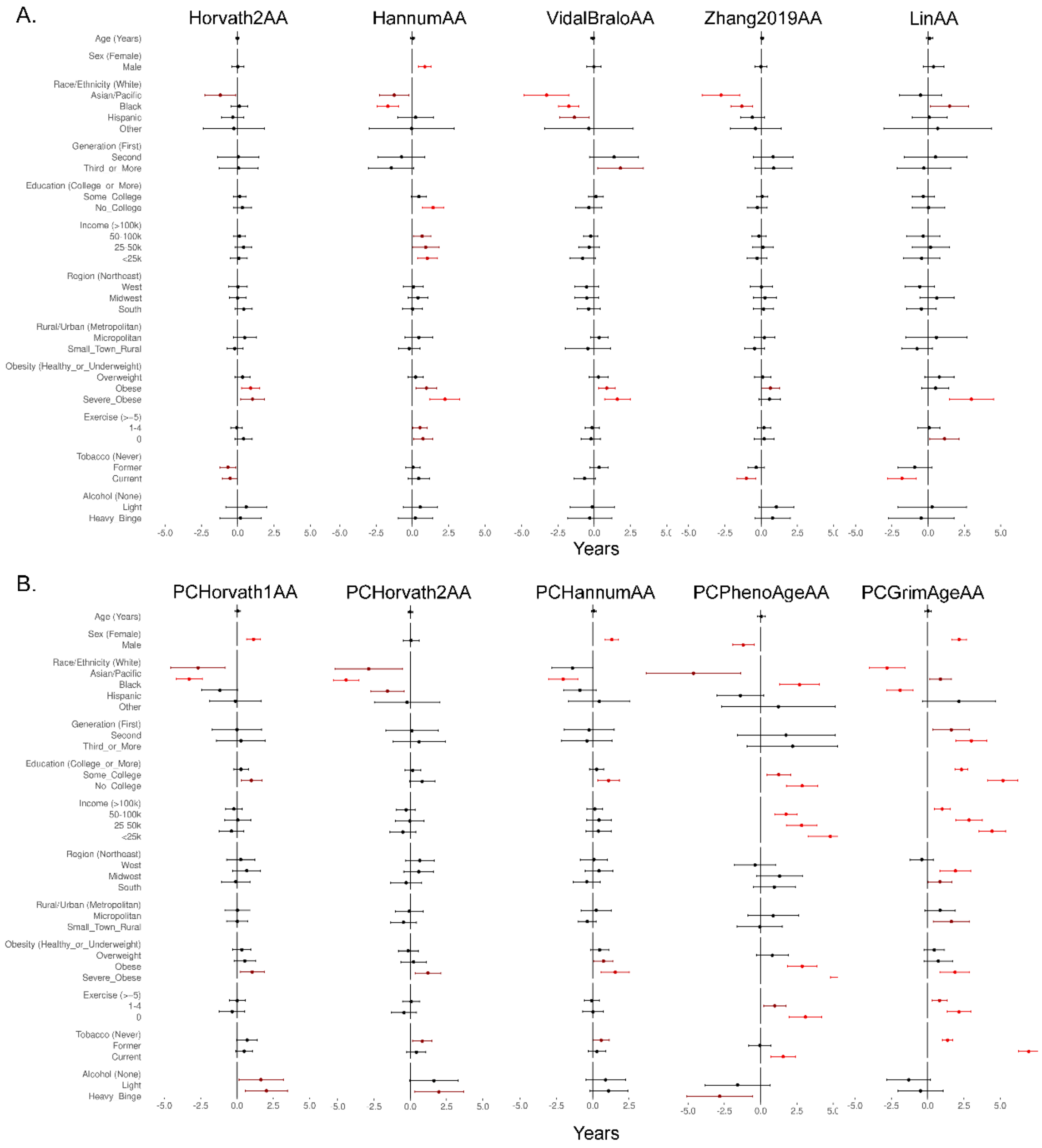

Weighted bivariate associations between epigenetic age acceleration and sociodemographic covariates are depicted in a forest plot. Red 95% confidence intervals around point estimates indicate statistical significance, while black intervals denote nonsignificance. The lightest red hues indicate p<0.0005, the middle red hues indicate 0.005<p<0.0005, and the darkest red hues indicate 0.05<p<0.005. The 'other' category for race/ethnicity encompasses individuals identifying as Native American or selecting 'other' for race/ethnicity, often indicating multiple races and/or ethnicities. Unless otherwise specified, age acceleration units are expressed in biological years. Panel A displays first and second-generation clocks not featured in Figure 2. Panel B showcases principal component clock acceleration. Panel C illustrates the rate adjustment associated with each sociodemographic covariate. Panel D presents the associated risk estimate for all-cause mortality from Zhang 2017.

© 2024 Harris KM et al. *JAMA Network Open.*

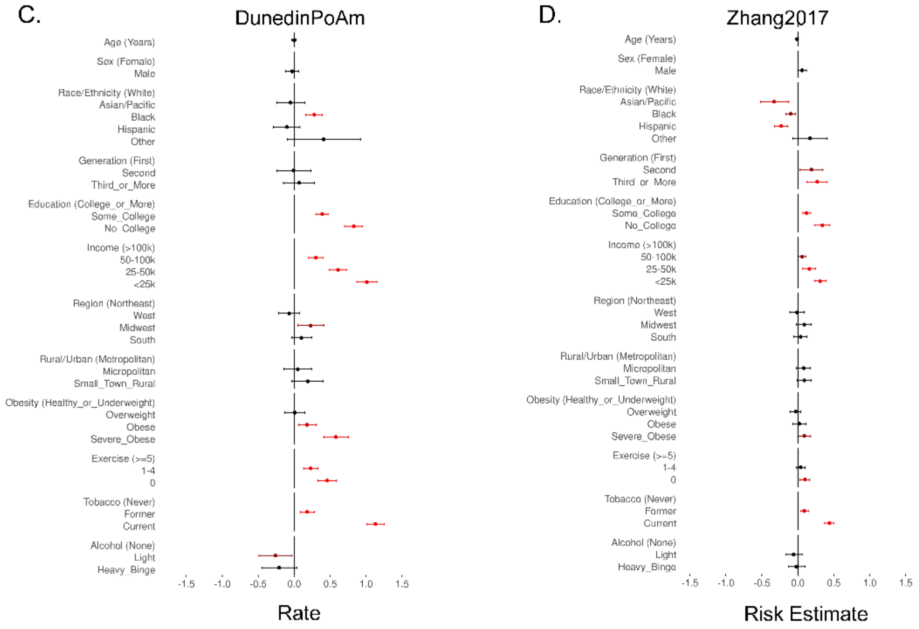

© 2024 Harris KM et al. *JAMA Network Open.*

eTable 1. Add Health sample distribution of demographic characteristics at Wave I, Wave V full sample, and Wave V Epigenetic sample.						
	Wave I (N=18,924)[a]		Wave V (N=11,718)[b]		Wave V Epigenetic sample (N=4237)[c]	
**WI variable**	**N**	**Weighted %**	**N**	**Weighted %**	**N**	**Weighted %**
**Race or ethnicity**						
Amer Indian or Alaska Native	233	1.11	100	0.897	17	0.58
Asian	1265	3.47	666	3.27	195	2.48
Black	4080	16.00	2323	15.92	811	16.75
Hispanic	2930	10.60	1452	9.39	435	8.70
Other[d]	68	0.23	48	0.34	10	0.26
Pacific Islander	97	0.26	97	0.46	23	0.26
White	10251	68.23	7032	69.71	2746	70.97
**Sex**						
Male	9290	50.92	5103	50.39	1671	48.83
Female	9634	49.08	6615	49.61	2566	51.17
**Immigrant Gen[e]**						
First	1561	5.66	770	5.15	200	4.36
Second	2795	10.75	1624	10.68	551	9.96
≥Third	14317	83.59	9178	84.17	3486	85.68

Notes:

[a] Weighted with Wave I sampling weights (participants missing sampling weights excluded, N=1821).

[b] Weighted with Wave V sampling weights.

[c] Weighted with Wave V biosample weights.

[d] The other category for race or ethnicity includes participants who identified as American Indian or Alaska Native or who checked some other race or origin.

[e] There were 252 missing on immigrant generation at Wave I and 152 missing on immigrant generation at Wave V.

© 2024 Harris KM et al. *JAMA Network Open.*

**eTable 2. Univariate Statistics for DNA Methylation Epigenetic Clocks (N=4237)**						
**Type of Measure**	**Measure**	**10%**	**25%**	**50%**	**75%**	**90%**
Chronological Age*	Years of Age	35.9	37.0	38.5	39.9	40.9
Clocks (years)	Horvath1	33.7	36.3	39.1	42.0	44.6
	Horvath2	31.1	33.2	35.4	37.6	39.8
	Hannum	26.6	28.8	31.5	34.0	36.2
	PhenoAge	22.8	26.3	30.1	33.8	37.3
	GrimAge	47.1	49.1	51.6	54.7	58.5
	Lin	17.0	20.0	23.3	26.6	29.8
	VidalBralo	50.5	52.5	54.7	57.1	59.4
	Zhang2019	26.9	29.3	32.0	34.5	37.0
PC Clocks (years)	PCHorvath1	40.4	42.7	45.1	47.6	49.9
	PCHorvath2	35.9	38.6	41.2	44.0	46.3
	PCHannum	41.1	43.5	46.1	48.6	50.8
	PCPhenoAge	34.5	37.6	40.9	44.3	47.9
	PCGrimAge	50.0	51.8	53.9	56.3	59.4
Age Acceleration (years)	Horvath1AA	-4.5	-1.7	0.0	1.6	4.1
	Horvath2AA	-3.1	-1.2	0.0	1.1	3.1
	HannumAA	-4.0	-1.7	-0.1	1.5	4.0
	PhenoAgeAA	-6.0	-2.4	0.0	2.2	6.0
	GrimAgeAA	-4.6	-2.4	-0.5	1.1	4.9
	LinAA	-5.6	-2.0	0.1	2.1	5.3
	VidalBraloAA	-3.8	-1.6	-0.1	1.3	3.8
	Zhang2019AA	-3.4	-1.5	-0.1	1.3	3.3
PC Age Acceleration (years)	PCHorvath1AA	-3.9	-1.6	-0.1	1.4	3.7
	PCHorvath2AA	-4.4	-1.7	0.0	1.7	4.2
	PCHannumAA	-4.0	-1.6	0.0	1.4	3.8
	PCPhenoAgeAA	-5.3	-2.2	-0.1	2.0	5.5
	PCGrimAgeAA	-3.8	-2.0	-0.5	0.8	3.7
Rate of Aging (SD)	Dunedin PoAm	-1.1	-0.6	-0.1	0.6	1.3
	Dunedin PACE	-1.2	-0.7	-0.1	0.6	1.3
Risk of mortality (RR)	Zhang2017	-1.8	-1.6	-1.3	-1.1	-0.8

Notes:

* Chronological age at Wave V blood draw.

© 2024 Harris KM et al. *JAMA Network Open.*
