# Supplementary material for: Sociodemographic and Lifestyle Factors and Epigenetic Aging in US Young Adults: NIMHD Social Epigenomics Program
Source: JAMA Netw Open. 2024 Jul 29;7(7):e2427889. doi: 10.1001/jamanetworkopen.2024.27889 (PMC11287395; doi:10.1001/jamanetworkopen.2024.27889)
Supplement: Supplement 3. — Data Sharing Statement [file jamanetwopen-e2427889-s003.pdf]

Data Sharing Statement

Harris. Sociodemographic and Lifestyle Factors and Epigenetic Aging in US Young Adults. *JAMA Netw Open*. Published online July 29, 2024. doi:10.1001/jamanetworkopen.2024.27889

Data
Data available: Yes
Data types: Deidentified participant data **How to access data:** kathie_harris@unc.edu **When available:** beginning date: 04-01-2025

Supporting Documents
Document types: None

Additional Information
Who can access the data: researchers who apply for a restricted data contract.
Types of analyses: Any research-based analysis
Mechanisms of data availability: With a signed data use agreement
